# Supplementary material for: SUSA2 is an F-box protein required for autoimmunity mediated by paired NLRs SOC3-CHS1 and SOC3-TN2
Source: Nat Commun. 2020 Oct 15;11:5190. doi: 10.1038/s41467-020-19033-z (PMC7562919; doi:10.1038/s41467-020-19033-z)
Supplement: Supplementary file 1 — Supplementary Information [file 41467_2020_19033_MOESM1_ESM.pdf]

Inventory of Supplementary Information of Liang et al.

SUSA2 is an F-box protein required for autoimmunity mediated by paired NLRs SOC3-CHS1 and SOC3-TN2

Wanwan Liang, Meixuezi Tong and Xin Li

Supplementary Figures

Supplementary Figure 1
Supplementary Figure 2
Supplementary Figure 3
Supplementary Figure 4
Supplementary Figure 5
Supplementary Figure 6
Supplementary Figure 7
Supplementary Figure 8
Supplementary Figure 9
Supplementary Figure 10
Supplementary Figure 11
Supplementary Figure 12
Supplementary Figure 13
Supplementary Figure 14
Supplementary Figure 15

Supplementary Tables

Supplementary Table 1

Supplementary Figures

Supplementary Figure 1

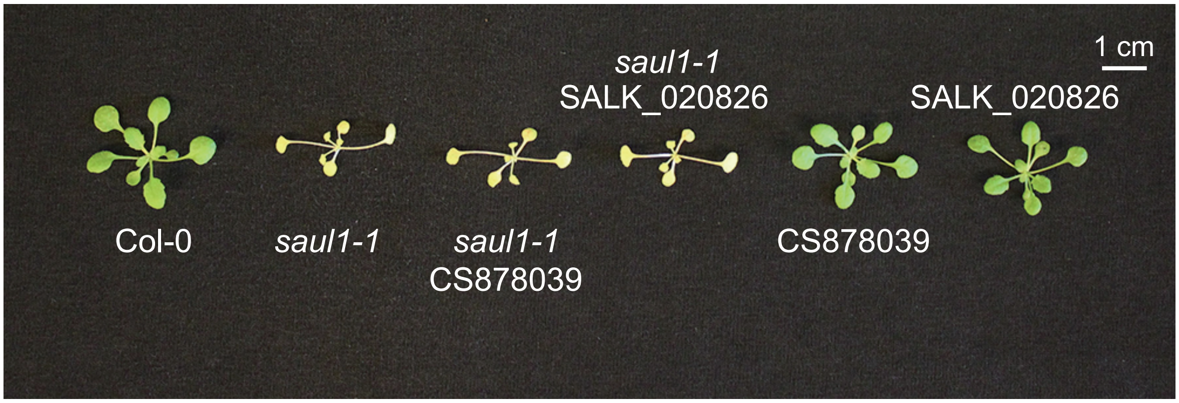

Supplementary Figure 1. CS878039 and SALK_020826 do not suppress the autoimmunity of *saul1-1*.

Morphology of 3.5-week-old Col-0, *saul1-1*, *saul1-1* CS878039 and *saul1-1* SALK_020826 double mutant plants. Plants were grown on 1/2 MS medium for 10 days and then transplanted to soil for another two weeks before the picture was taken.

Supplementary Figure 2

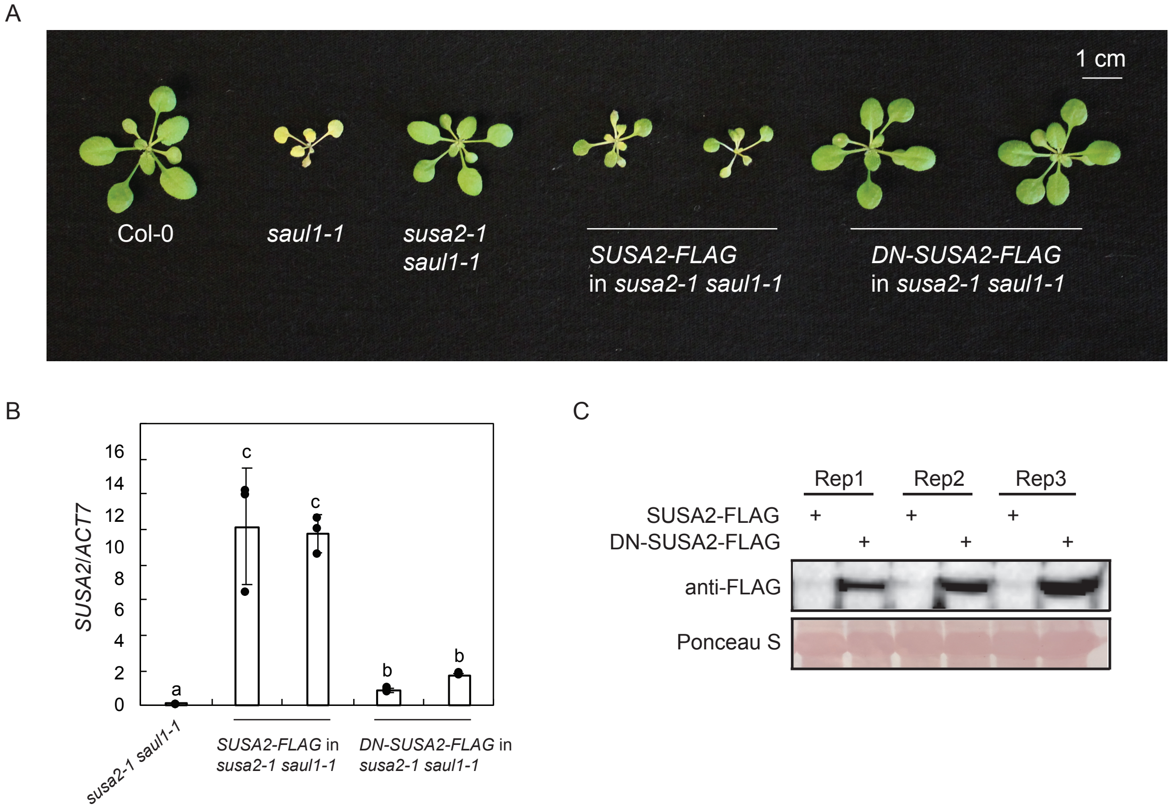

Supplementary Figure 2. F-box deletion increases SUSA2 protein abundance.

(A) Morphology of 4-week-old Col-0, *saul1-1*, *susa2-1 saul1-1*, two transgenic lines of *SUSA2-FLAG* in *susa2-1 saul1-1* and two transgenic lines of *DN-SUSA2-FLAG* in *susa2-1 saul1-1*.

(B) *SUSA2* expression in the indicated plants in (A) as determined by RT-PCR and normalized to *ACT7*. Error bars represent means ±SD (One-way ANOVA, SPSS Statistics, n=3, $p<0.01$). Experiments were repeated three times with similar results.

(C) Protein expression of SUSA2-FLAG and DN-SUSA2-FLAG in *N. benthamiana*. Agrobacteria carrying *SUSA2-FLAG* or *DN-SUSA2-FLAG* construct was infiltrated into the left half or right half of *N. benthamiana* leaves, respectively, to be expressed for 48 hr before harvested for western blot analysis. Three replicates (rep) were performed.

Supplementary Figure 3

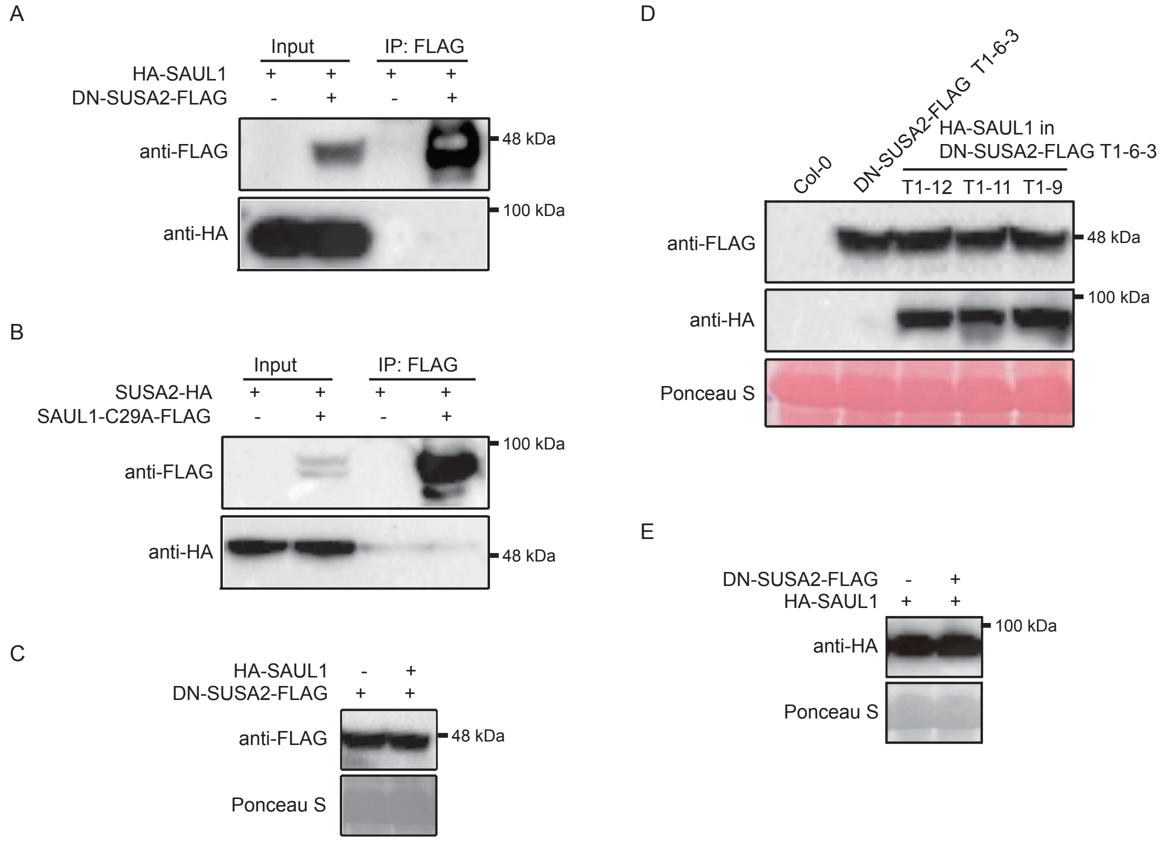

Supplementary Figure 3. SUSA2 and SAUL1 do not associate with each other, and DN-SUSA2-FLAG and HA-SAUL1 has no effect on each other's protein abundance.

(A) Co-immunoprecipitation analysis of SUSA2 and SAUL1. Agrobacteria carrying *DN-SUSA2-FLAG* with *HA-SAUL1* constructs or empty vector (-) with *HA-SAUL1* constructs were co-infiltrated into *N. benthamiana* leaves. After 48 hr incubation, leaf tissue was harvested for co-immunoprecipitation analysis.

(B) Co-immunoprecipitation analysis of SUSA2 and SAUL1. Agrobacteria carrying *SAUL1-C29A-FLAG* with *SUSA2-HA* constructs or empty vector (-) with *SUSA2-HA* constructs were co-infiltrated into *N. benthamiana* leaves. After 48 hr incubation, leaf tissue was harvested for co-immunoprecipitation analysis.

(C) HA-SAUL1 has no effect on DN-SUSA2-FLAG protein abundance. Agrobacteria carrying *DN-SUSA2-FLAG* and *HA-SAUL1* constructs or empty vector (-) were co-infiltrated into *N.*

benthamiana leaves. After 48 hr incubation, leaf tissue was harvested for western blot analysis.
(D) HA-SAUL1 has no effect on DN-SUSA2-FLAG protein abundance in Arabidopsis. HA-SAUL1 was transformed in DN-SUSA2-FLAG T1-6-3 transgenic line. Three transformants with high HA-SAUL1 expression were selected for testing the effect of SAUL1 overexpression on DN-SUSA2-FLAG protein level.
(E) DN-SUSA2-FLAG has no effect on HA-SAUL1 protein abundance. Agrobacteria carrying *HA-SAUL1* and *DN-SUSA2-FLAG* constructs or empty vector (-) were co-infiltrated into *N. benthamiana* leaves. After 48 hr incubation, leaf tissue was harvested for western blot analysis.

Supplementary Figure 4

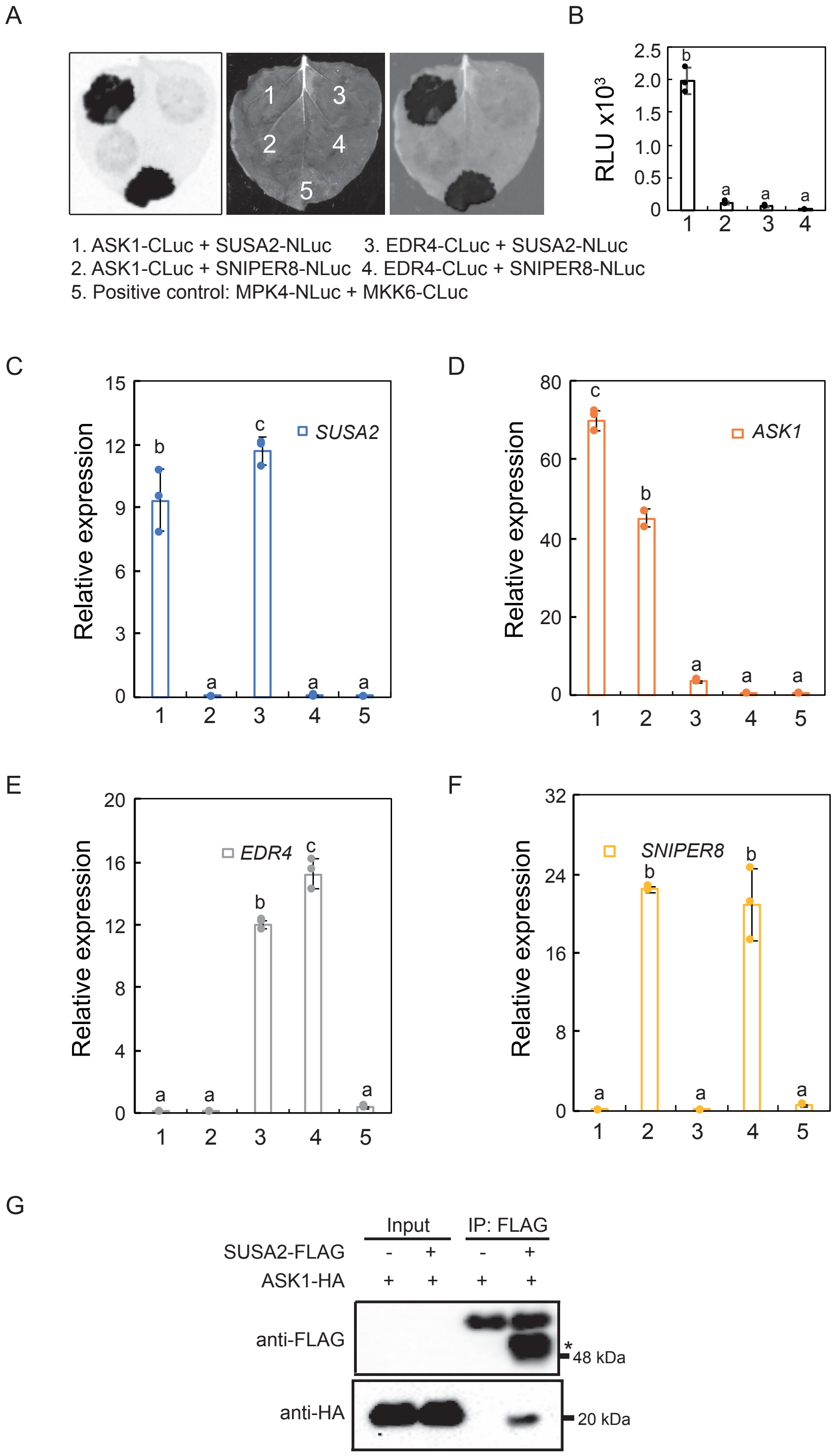

Supplementary Figure 4. SUSA2 associates with ASK1 in split-luciferase assay.

(A) SUSA2 and ASK1 interaction as revealed in Split-Luciferase assays. Agrobacteria carrying *ASK1-cLuc* and *SUSA2-nLuc* constructs or other indicated paired constructs were co-infiltrated into *N. benthamiana* leaves at OD_{600} = 0.2. The photo was taken 48 hr post infiltration. MPK4-NLuc and MKK6-CLuc serve as the positive control. ENHANCED DISEASE RESISTANCE4 (EDR4) is involved in negative regulation of resistance to powdery mildew [70]. SNIPER8 is an immune-regulating E3 ligase isolated from *snc1*-influencing plant E3 ligase reverse (SNIPER) genetic screen, the data of which has not been published (Paul Kapos and Xin Li).

(B) Quantification of chemiluminescence in (A). The luminescence of positive control was too high (6002 ± 873) and it masked the relatively weak interaction intensity between ASK1 and SUSA2. It was therefore not included. Error bars represent means ±SD (One-way ANOVA, SPSS Statistics, n=3, $p<0.01$). Experiments were repeated three times with similar results.

(C) -(F) Expression level of *SUSA2*, *ASK1*, *EDR4* and *SNIPER8* in the indicated infiltrated area in (A) as determined by RT-PCR and normalized to *NbACTIN* gene. Error bars represent means ±SD (One-way ANOVA, SPSS Statistics, n=3, $p<0.01$). Experiments were repeated three times with similar results.

(G) SUSA2-FLAG is able to pull down ASK1-HA in Arabidopsis Col-0 stable transgenic lines with both transgenes.

Supplementary Figure 5

```
ARP3          1 -------------------------------------------------------MDPTS
ARP6          1 -----------------------------------------------------------M
ACT2          1 ------------------------------------------------------MAEADD
ACT8          1 ------------------------------------------------------MADADD
ACT1          1 ------------------------------------------------------MADGED
ACT3          1 ------------------------------------------------------MADGED
ACT12         1 ------------------------------------------------------MADGED
ACT4          1 ------------------------------------------------------MADGED
ACT11         1 ------------------------------------------------------MADGED
ACT7          1 ------------------------------------------------------MADGED
At2g42100     1 -----------------------------------------------------MSDLGDE
At2g42170     1 ------------------------------------------------------------
ACT9          1 ------------------------------------------------------------
ARP2          1 ---------------------------------------------------------MDN
ARP4          1 ------------------------------------------------------MYGGDE
ARP7          1 ------------------------------------------------------------
ARP5          1 ------------------------------------------MAFVSRIRRQSDYNTYPS
SUSA2         1 MILKKVWGSVWNRSNSGKDLVNHQRAIDVPPLLLSSSSSLGAFDQLPMDILVQILMMMEP


ARP3          6 RPAIVIDNGTGYTKMGFAG--------------------------NVEPCFILPTVVAVN
ARP6          2 SNIVVLDNGGGLIKAGQGG--------------------------ERDPTTVIPNCLYKP
ACT2          7 IQPIVCDNGTGMVKAGFAG--------------------------DDAPRAVFPSVVGR-
ACT8          7 IQPIVCDNGTGMVKAGFAG--------------------------DDAPRAVFPSVVGR-
ACT1          7 IQPIVCDNGTGMVKAGFAG--------------------------DDAPRAVFPSIVGR-
ACT3          7 IQPIVCDNGTGMVKAGFAG--------------------------DDAPRAVFPSIVGR-
ACT12         7 IQPIVCDNGTGMVKAGFAG--------------------------DDAPRAVFPSIVGR-
ACT4          7 IQPIVCDNGTGMVKAGFAG--------------------------DDAPRAVFPSIVGR-
ACT11         7 IQPIVCDNGTGMVKAGFAG--------------------------DDAPRAVFPSIVGR-
ACT7          7 IQPIVCDNGTGMVKAGFAG--------------------------DDAPRAVFPSIVGR-
At2g42100     8 SVAIVCDNGTGMVKAGFAG--------------------------DDAPRAVFPSVVGR-
At2g42170     1 ------------------------------------------------------------
ACT9          1 MKPIVCDKGHGMVQAGFAG--------------------------DEAPKVVFPCVVGR-
ARP2          4 KNVVVCDNGTGYVKCGFAG--------------------------ENFPTSVFPCVVGRP
ARP4          7 VSAIVVDLGSHTCKAGYAG--------------------------EDAPKAVFPSVIGAV
ARP7          1 MEALVVDAGSKFLKAGAAIP-------------------------DQSPAMIIPSQMKR-
ARP5         19 SIPIVIDNGASYFRIGWAG--------------------------ETEPRVVFRNIVQRP
SUSA2        61 KDAVKLGLTCKAWKCVASGNRLWIFYLQCSQEPWDSIFFAETSLRSGYPLRMISSQSGEL


ARP3         40 ESF----LNQSKSSSKATWQTQHNAGVAADLDFYIGDEALAKSRSSSTHNLHYPIEHGQV
ARP6         36 LSS----KKFIHPSP---------------------LTTLSDEIDLTSAAVRRPIDRGYL
ACT2         40 --------------------PRHHGVMVGMNQKDAYVGDEAQSKR-GILTLKYPIEHGVV
ACT8         40 --------------------PRHHGVMVGMNQKDAYVGDEAQSKR-GILTLKYPIEHGVV
ACT1         40 --------------------PRHTGVMVGMGQKDAYVGDEAQSKR-GILTLKYPIEHGIV
ACT3         40 --------------------PRHTGVMVGMGQKDAYVGDEAQSKR-GILTLKYPIEHGIV
ACT12        40 --------------------PRHTGVMVGMGQKDAYVGDEAQSKR-GILTLKYPIEHGIV
ACT4         40 --------------------PRHTGVMVGMGQKDAYVGDEAQSKR-GILTLKYPIEHGIV
ACT11        40 --------------------PRHTGVMVGMGQKDAYVGDEAQSKR-GILTLKYPIEHGIV
ACT7         40 --------------------PRHTGVMVGMGQKDAYVGDEAQSKR-GILTLKYPIEHGIV
At2g42100    41 --------------------PRHRGVMVGMDEKDTFVGDEAQARR-GILSLKYPIEHGVV
At2g42170     1 --------------------------MVGMNENDLFVGDDAEARS-GILTLDYPMEHGVV
ACT9         34 --------------------PRD-----GLNPNESYVGEEGHANR-DILTLKDPMEHGIV
ARP2         38 L-------------------LRYEESLMEQQVKDIVVGETCSELR-HQLDINYPVHNGIV
ARP4         41 DGVEAMDVDVDSTKTNSNSEDSKTESEKEKSKRKLYVGSQAMSYRRDHMEVLSPIKDGIV
ARP7         35 -------------------------------MVDDGSSSADNPTTVFEDVTLDPIERGLI
ARP5         53 R---------------HKATVLLSSTGETVTIVGDLDPSMMKYFDCTRSGPRSPFDSNVV
SUSA2       121 SFMHIYSQRAQVPG---SIIIDGGSGYCKFGWSKYASPSGRSATFLEFGNIESPIYARLQ
                                                                     *
```

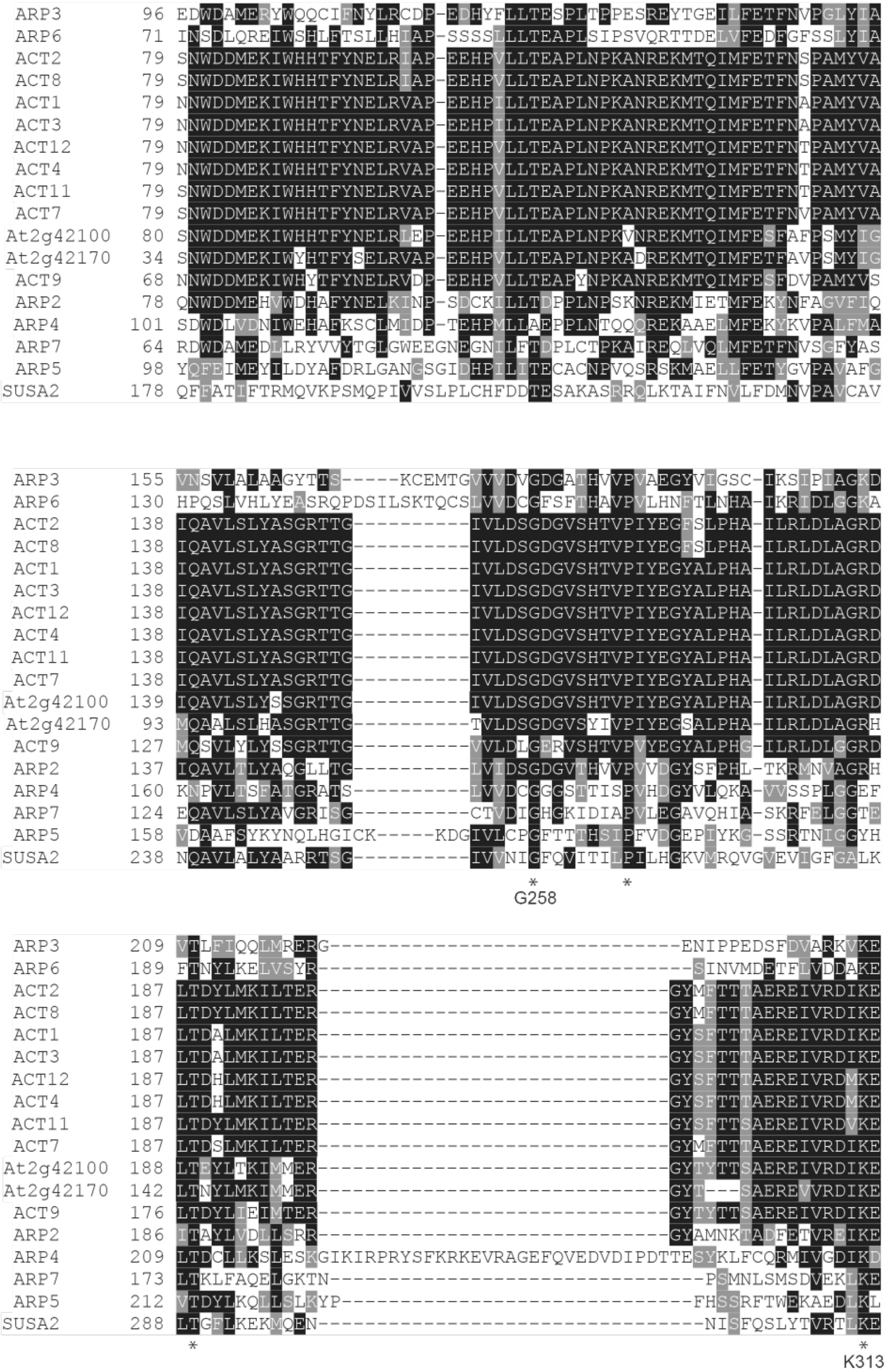
ARP3 96 EDWDAMERYWQQCIFNYLRCDP-EDHYFLLTESPLTPPESREYTGEILFETFNVPGLYIA
ARP6 71 INSDLQREIWSHLFTSLLHIAP-SSSSLLLTEAPLSIPSVQRTTDELVFEDFGFSSLYIA
ACT2 79 SNWDDMEKIWHHTFYNELRIAP-EEHPVLLTEAPLNPKANREKMTQIMFETFNSPAMYVA
ACT8 79 SNWDDMEKIWHHTFYNELRIAP-EEHPVLLTEAPLNPKANREKMTQIMFETFNSPAMYVA
ACT1 79 NNWDDMEKIWHHTFYNELRVAP-EEHPILLTEAPLNPKANREKMTQIMFETFNAPAMYVA
ACT3 79 NNWDDMEKIWHHTFYNELRVAP-EEHPILLTEAPLNPKANREKMTQIMFETFNAPAMYVA
ACT12 79 NNWDDMEKIWHHTFYNELRVAP-EEHPVLLTEAPLNPKANREKMTQIMFETFNTPAMYVA
ACT4 79 NNWDDMEKIWHHTFYNELRVAP-EEHPVLLTEAPLNPKANREKMTQIMFETFNTPAMYVA
ACT11 79 SNWDDMEKIWHHTFYNELRVAP-EEHPVLLTEAPLNPKANREKMTQIMFETFNTPAMYVA
ACT7 79 SNWDDMEKIWHHTFYNELRVAP-EEHPVLLTEAPLNPKANREKMTQIMFETFNVPAMYVA
At2g42100 80 SNWDDMEKIWHHTFYNELRIEP-EEHPILLTEAPLNPKVNREKMTQIMFESFAFPSMYIG
At2g42170 34 SNWDDMEKIWYHTFYSELRVAP-EEHPVLLTEAPLNPKADREKMTQIMFETFAVPSMYIG
ACT9 68 NNWDDMEKIWHYTFYNELRVDP-EEHPVLLTEAPYNPKANREKMTQIMFESFDVPAMYVS
ARP2 78 QNWDDMEHVWDHAFYNELKINP-SDCKILLTDPPLNPSKNREKMIETMFEKYNFAGVFIQ
ARP4 101 SDWDLVDNIWEHAFKSCLMIDP-TEHPMLLAEPPLNTQQQREKAAELMFEKYKVPALFMA
ARP7 64 RDWDAMEDLLRYVVYTGLGWEEGNEGNILFTDPLCTPKAIREQLVQLMFETFNVSGFYAS
ARP5 98 YQFEIMEYILDYAFDRLGANGSGIDHPILITECACNPVQSRSKMAELLFETYGVPAVAFG
SUSA2 178 QFFATIFTRMQVKPSMQPIVVSLPLCHFDDTESAKASRRQLKTAIFNVLFDMNVPAVCAV
ARP3 155 VNSVLALAAGYTTS-----KCEMTGVVVDVGDGATHVVPVAEGYVIGSC-IKSIPIAGKD
ARP6 130 HPQSLVHLYEASRQPDSILSKTQCSLVVDCGFSFTHAVPVLHNFTLNHA-IKRIDLGGKA
ACT2 138 IQAVLSLYASGRTTG----------IVLDSGDGVSHTVPIYEGFSLPHA-ILRLDLAGRD
ACT8 138 IQAVLSLYASGRTTG----------IVLDSGDGVSHTVPIYEGFSLPHA-ILRLDLAGRD
ACT1 138 IQAVLSLYASGRTTG----------IVLDSGDGVSHTVPIYEGYALPHA-ILRLDLAGRD
ACT3 138 IQAVLSLYASGRTTG----------IVLDSGDGVSHTVPIYEGYALPHA-ILRLDLAGRD
ACT12 138 IQAVLSLYASGRTTG----------IVLDSGDGVSHTVPIYEGYALPHA-ILRLDLAGRD
ACT4 138 IQAVLSLYASGRTTG----------IVLDSGDGVSHTVPIYEGYALPHA-ILRLDLAGRD
ACT11 138 IQAVLSLYASGRTTG----------IVLDSGDGVSHTVPIYEGYALPHA-ILRLDLAGRD
ACT7 138 IQAVLSLYASGRTTG----------IVLDSGDGVSHTVPIYEGYALPHA-ILRLDLAGRD
At2g42100 139 IQAVLSLYSSGRTTG----------IVLDSGDGVSHTVPIYEGYALPHA-ILRLDLAGRD
At2g42170 93 MQAALSLHASGRTTG----------TVLDSGDGVSYIVPIYEGSALPHA-ILRLDLAGRH
ACT9 127 MQSVLYLYSSGRTTG----------VVLDLGERVSHTVPVYEGYALPHG-ILRLDLGGRD
ARP2 137 IQAVLTLYAQGLLTG----------LVIDSGDGVTHVVPVVDGYSFPHL-TKRMNVAGRH
ARP4 160 KNPVLTSFATGRATS----------LVVDCGGGSTTISPVHDGYVLQKA-VVSSPLGGEF
ARP7 124 EQAVLSLYAVGRISG----------CTVDIGHGKIDIAPVLEGAVQHIA-SKRFELGGTE
ARP5 158 VDAAFSYKYNQLHGICK-----KDGIVLCPGFTTTHSIPFVDGEPIYKG-SSRTNIGGYH
SUSA2 238 NQAVLALYAARRTSG----------IVVNIGFQVITILPILHGKVMRQVGVEVIGFGALK
* *
G258
ARP3 209 VTLFIQQLMRERG------------------------------ENIPPEDSFDVARKVKE
ARP6 189 FTNYLKELVSYR--------------------------------SINVMDETFLVDDAKE
ACT2 187 LTDYLMKILTER------------------------------GYMFTTTAEREIVRDIKE
ACT8 187 LTDYLMKILTER------------------------------GYMFTTTAEREIVRDIKE
ACT1 187 LTDALMKILTER------------------------------GYSFTTTAEREIVRDIKE
ACT3 187 LTDALMKILTER------------------------------GYSFTTTAEREIVRDIKE
ACT12 187 LTDHLMKILTER------------------------------GYSFTTTAEREIVRDMKE
ACT4 187 LTDHLMKILTER------------------------------GYSFTTTAEREIVRDMKE
ACT11 187 LTDYLMKILTER------------------------------GYSFTTSAEREIVRDVKE
ACT7 187 LTDSLMKILTER------------------------------GYMFTTTAEREIVRDIKE
At2g42100 188 LTEYLTKIMMER------------------------------GYTYTTSAEREIVRDIKE
At2g42170 142 LTNYLMKIMMER------------------------------GYT---SAEREVVRDIKE
ACT9 176 LTDYLIEIMTER------------------------------GYTYTTSAEREIVRDIKE
ARP2 186 ITAYLVDLLSRR------------------------------GYAMNKTADFETVREIKE
ARP4 209 LTDCLLKSLESKGIKIRPRYSFKRKEVRAGEFQVEDVDIPDTTESYKLFCQRMIVGDIKD
ARP7 173 LTKLFAQELGKTN--------------------------------PSMNLSMSDVEKLKE
ARP5 212 VTDYLKQLLSLKYP------------------------------FHSSRFTWEKAEDLKL
SUSA2 288 LTGFLKEKMQEN---------------------------------NISFQSLYTVRTLKE
* *
K313

```
ARP3       239 MYCYTCSDIVKEFNKHD-------------------------------------------
ARP6       217 KLCFVSLDLLRDLRLARNGNT---------------------------------------
ACT2       217 KLSFVAVDYEQEMETSKTSS----------------------------------------
ACT8       217 KLSFVAVDYEQEMETSKTSS----------------------------------------
ACT1       217 KLCYIALDYEQELETAKTSS----------------------------------------
ACT3       217 KLCYIALDYEQELETAKTSS----------------------------------------
ACT12      217 KLSYIALDYEQELETSKTSS----------------------------------------
ACT4       217 KLSYIALDFEQELETSKTSS----------------------------------------
ACT11      217 KLAYIALDYEQEMETANTSS----------------------------------------
ACT7       217 KLAYVALDYEQELETAKSSS----------------------------------------
At2g42100  218 KLCYIAVDYEQEMEKATTSS----------------------------------------
At2g42170  169 QFGYIALDYEQEMEKATKSS----------------------------------------
ACT9       206 KLCYVALDYEQEMEKTTKGW----------------------------------------
ARP2       216 KLCYISYDYKRESQLGLETT----------------------------------------
ARP4       269 SICRVPDTPYDD---KSYSN----------------------------------------
ARP7       201 QYANCAEDEIAYKKTQN-------------------------------------------
ARP5       242 EHCYIAPDYASEIRLFQEGRKEAEEKTSYWQLPWIPPPTEVPPSEEEIARKAAIREKQGQ
SUSA2      315 KLCYVALDYKAELSKDTQAS----------------------------------------
```

```
ARP3       256 ------------------------------------------------------------
ARP6       238 ------------------------------------------------------------
ACT2       237 ------------------------------------------------------------
ACT8       237 ------------------------------------------------------------
ACT1       237 ------------------------------------------------------------
ACT3       237 ------------------------------------------------------------
ACT12      237 ------------------------------------------------------------
ACT4       237 ------------------------------------------------------------
ACT11      237 ------------------------------------------------------------
ACT7       237 ------------------------------------------------------------
At2g42100  238 ------------------------------------------------------------
At2g42170  189 ------------------------------------------------------------
ACT9       226 ------------------------------------------------------------
ARP2       236 ------------------------------------------------------------
ARP4       286 ------------------------------------------------------------
ARP7       218 ------------------------------------------------------------
ARP5       302 RLREMAEAKRVSKINDMENQLISLRFLLKQVDQVEEDDIPTFLSDTGYASRQELESTITK
SUSA2      335 ------------------------------------------------------------
```

```
ARP3       256 ------------------------------------------------------------
ARP6       238 ------------------------------------------------------------
ACT2       237 ------------------------------------------------------------
ACT8       237 ------------------------------------------------------------
ACT1       237 ------------------------------------------------------------
ACT3       237 ------------------------------------------------------------
ACT12      237 ------------------------------------------------------------
ACT4       237 ------------------------------------------------------------
ACT11      237 ------------------------------------------------------------
ACT7       237 ------------------------------------------------------------
At2g42100  238 ------------------------------------------------------------
At2g42170  189 ------------------------------------------------------------
ACT9       226 ------------------------------------------------------------
ARP2       236 ------------------------------------------------------------
ARP4       286 ------------------------------------------------------------
ARP7       218 ------------------------------------------------------------
ARP5       362 VTQSLRKARGEPKNEPAEYEENPDSLNNEKYPLMNVPDDILTPEQLKDKKRQMFLKTTAE
SUSA2      335 ------------------------------------------------------------
```

```
ARP3       256 ------------------------------------------------------------
ARP6       238 ------------------------------------------------------------
ACT2       237 ------------------------------------------------------------
ACT8       237 ------------------------------------------------------------
ACT1       237 ------------------------------------------------------------
ACT3       237 ------------------------------------------------------------
ACT12      237 ------------------------------------------------------------
ACT4       237 ------------------------------------------------------------
ACT11      237 ------------------------------------------------------------
ACT7       237 ------------------------------------------------------------
At2g42100  238 ------------------------------------------------------------
At2g42170  189 ------------------------------------------------------------
ACT9       226 ------------------------------------------------------------
ARP2       236 ------------------------------------------------------------
ARP4       286 ------------------------------------------------------------
ARP7       218 ------------------------------------------------------------
ARP5       422 GRLRARQKRNEEELEKEKRNQLEEERRRENPESYLEELQAQYKEVLERVEQKKRLKTNGS
SUSA2      335 ------------------------------------------------------------
```

```
ARP3       256 ------------------------------------------------------------
ARP6       238 ----------------------------------------------------------LI
ACT2       237 ------------------------------------------------------------
ACT8       237 ------------------------------------------------------------
ACT1       237 ------------------------------------------------------------
ACT3       237 ------------------------------------------------------------
ACT12      237 ------------------------------------------------------------
ACT4       237 ------------------------------------------------------------
ACT11      237 ------------------------------------------------------------
ACT7       237 ------------------------------------------------------------
At2g42100  238 ------------------------------------------------------------
At2g42170  189 ------------------------------------------------------------
ACT9       226 ------------------------------------------------------------
ARP2       236 ------------------------------------------------------------
ARP4       286 ------------------------------------------------------------
ARP7       218 ------------------------------------------------------------
ARP5       482 SNGNNKSGGIGRGERLSAAQRERMRLLTTAAFDRGKGEDTFGSRDEDWQLYKLMSKDNDD
SUSA2      335 ------------------------------------------------------------
```

```
ARP3       256 -------------------------------KEPAKYIKQWKGVKPKTGAPYTCDVGYER
ARP6       240 KSTYVLPDGVTHTKGYVKDPQAAKRFLSLSEKESVVVMDKVGERKKADMNKNEIDLTNER
ACT2       237 ----------------------------------------SIEKNYELPDGQVITIGAER
ACT8       237 ----------------------------------------SIEKNYELPDGQVITIGAER
ACT1       237 ----------------------------------------SVEKNYELPDGQVITIGSER
ACT3       237 ----------------------------------------SVEKNYELPDGQVITIGSER
ACT12      237 ----------------------------------------SVEKSFELPDGQVITIGAER
ACT4       237 ----------------------------------------SVEKSFELPDGQVITIGAER
ACT11      237 ----------------------------------------SVEKSYELPDGQVITIGGER
ACT7       237 ----------------------------------------SVEKNYELPDGQVITIGAER
At2g42100  238 ----------------------------------------AIDRTYELPDGQVITIGAER
At2g42170  189 ----------------------------------------AIDRTYELPDGQVITIGAER
ACT9       226 ----------------------------------------TIDKTYVLPDGQEITIEAER
ARP2       236 ----------------------------------------ILVKNYTLPDGRVIKVGTER
ARP4       286 ----------------------------------------IPTTSYELPDGQTLEIGADR
ARP7       218 ----------------------------------------CEIEQHTLPDGQVISIGRER
ARP5       542 DDEQPDSDEAELARLSSRLQEIDPTFVQKVEGELSQTSGEVPRVRPLTEEDYKIVIGIER
SUSA2      335 ---------------------------------------------VEVSGEGWFTLSKER
                                                                          *
```

```
ARP3       285 FLGPEVFFNPEIYSNDFTTT-----------LPAVIDKCIQSAPIDTRRALYKNIVLSGG
ARP6       300 FLVPETLFQPADLG-MNQAG-----------LAECIVRAINSCHSYLQPVLYQSIILTGG
ACT2       257 FRCPEVLFQPSFVGMEA------------AGIHETTYNSIMKCDVDIRKDLYGNIVLSGG
ACT8       257 FRCPEVLFQPSFVGMEA------------AGIHETTYNSIMKCDVDIRKDLYGNIVLSGG
ACT1       257 FRCPEVLYQPSMIGMEN------------AGIHETTYNSIMKCDVDIRKDLYGNIVLSGG
ACT3       257 FRCPEVLYQPSMIGMEN------------AGIHETTYNSIMKCDVDIRKDLYGNIVLSGG
ACT12      257 FRCPEVLFQPSMIGMEN------------PGIHETTYNSIMKCDVDIRKDLYGNIVLSGG
ACT4       257 FRCPEVLFQPSMIGMEN------------PGIHETTYNSIMKCDVDIRKDLYGNIVLSGG
ACT11      257 FRCPEVLFQPSLVGMEA------------AGIHETTYNSIMKCDVDIRKDLYGNIVLSGG
ACT7       257 FRCPEVLFQPSLIGMEA------------PGIHETTYNSIMKCDVDIRKDLYGNIVLSGG
At2g42100  258 FRCPEVLFQTSLIGMET------------SGIHETTYNSIMKCDVDIRKDLYGNIVLSGG
At2g42170  209 FRCPEVLFQPSLIGMET------------SGIHEKTYNSIMKCDDDIRKDLYGNIVLSGG
ACT9       246 FMCPEVLFQPSVIGKES------------SGIHEATRNSILKCPVDTRRDMYGNILMTGG
ARP2       256 FQAPEALFTPELIDVEG------------DGMADMVFRCIQEMDIDNRMMLYQHIVLSGG
ARP4       306 FKVPDVMFNPSIVQTIPGMEKYAEMIPSVRGLPHMVMESINKCDVDIRRELYSSILLAGG
ARP7       238 YSVGEALFQPSILGLEEHG-----------IVEQLVRIISTVSSENHRQLLENTVLCGG
ARP5       602 FRCPEILFHPNLIGIDQVG---------LDEMAGTSIRRLPHDEKELEERLTSSILMTGG
SUSA2      350 FQTGEILFQPRLAGMRAMS---------LHQAVSLCMDHCDAAGLTGDDSWFKTVVLTGG
                                                                         **
                                                                     G399 G400

ARP3       334 STMFKDFGRRLQRDLKKIVDARVLANNARTGGEITSQPVEVNVVSH---PVQRFAVWFGG
ARP6       348 STLFPQLKERLEGELRPLVPD---------------HFDVKITTQ---EDPILGVWRGG
ACT2       305 TTMFSGIADRMSKEITALAPS----------------SMKIKVVAP---PERKYSVWIGG
ACT8       305 TTMFSGIADRMSKEITALAPS----------------SMKIKVVAP---PERKYSVWIGG
ACT1       305 TTMFPGIADRMSKEITALAPS----------------SMKIKVVAP---PERKYSVWIGG
ACT3       305 TTMFPGIADRMSKEITALAPS----------------SMKIKVVAP---PERKYSVWIGG
ACT12      305 TTMFGGIGDRMSKEITALAPS----------------SMKIKVVAP---PERKYSVWIGG
ACT4       305 TTMFGGIGDRMSKEITALAPS----------------SMKIKVVAP---PERKYSVWIGG
ACT11      305 TTMFPGIADRMSKEITALAPS----------------SMKIKVVAP---PERKYSVWIGG
ACT7       305 STMFPGIADRMSKEITALAPS----------------SMKIKVVAP---PERKYSVWIGG
At2g42100  306 TTMFPGIADRMNKEINALAPP----------------SMKIKVVAP---PERKYSVWVGG
At2g42170  257 TTMFRGIEERMTKEINALAAA----------------NMRIKIVAP---PERKYSVWIGG
ACT9       294 TTMLHGIKERMTKELNALVPS----------------SMKVKVVVP---PESECSVWIGG
ARP2       304 STMYPGLPSRLEKEIQDRYLDTVLKGNKDG-----LKKLRLRIEDP---PRRKHMVYLGG
ARP4       366 TSSMQQLKERLEKDLIEESPH----------------SARVKVLASGNTTERRFSVWIGG
ARP7       286 TTSMTGFESRFQKEANLCSSAIR--------------PTLVKPPEYMPENLGMYSAWVGG
ARP5       653 CSLLPGMNERLECGIRMIRPCG----------------SPINVVRA---MDPVLDAWRGA
SUSA2      401 SACLPGISERLERELQDHLPSS--------------ISNGIRVIPP---PYGVDTSWHGA
                          *                                            *

ARP3       391 SVLSSTP-EFFASCRTKEEYEEYGASICRTNPVFKGMY
ARP6       389 SLLASSP-DFESMCVTKAEYEELGSARCRRRFH----
ACT2       346 SILASLS-TFQQMWISKAEYDEAGPGIVHRKCF-----
ACT8       346 SILASLS-TFQQMWISKAEYDEAGPGIVHRKCF-----
ACT1       346 SILASLS-TFQQMWIAKAEYDESGPSIVHRKCF-----
ACT3       346 SILASLS-TFQQMWIAKAEYDESGPSIVHRKCF-----
ACT12      346 SILASLS-TFQQMWIAKAEYDESGPSIVHRKCF-----
ACT4       346 SILASLS-TFQQMWIAKAEYDESGPSIVHRKCF-----
ACT11      346 SILASLS-TFQQMWIAKAEYDESGPSIVHRKCF-----
ACT7       346 SILASLS-TFQQMWISKSEYDESGPSIVHRKCF-----
At2g42100  347 SILASLS-SFAPMWITKAEYDEQGGAIVHRKCF-----
At2g42170  298 SILASLS-TYEQMWITKAEYEENGPAIVHTKCF-----
ACT9       335 SILASLS-TFHQMWITKDEYEEHGAAIVHRKCV-----
ARP2       356 AVLAGIMKDAPEFWINREDYMEEGINCLNKMSQA----
ARP4       410 SILASLG-SFQQMWFSKSEYEEHGASYIQRKCP-----
ARP7       332 AILAKVV-FPQNQHVTKADYDETGPSVVHRKCF-----
ARP5       694 SAFAANL-NFLGNAFTKMDYDEKGEDWLRNYQIRYNYL
SUSA2      444 KLISNLSIFPGPWCITRKQFRRKSRLMW----------
```

Supplementary Figure 5. Amino acid alignment of SUSA2, other ARPs and selected conventional ACTINs in Arabidopsis.

The protein sequences of SUSA2, other ARPs and selected conventional ACTINs were obtained from TAIR website by performing BLAST analysis using SUSA2 protein sequence as input. Sequences were aligned using Clustal1.83 and shaded with BOXSHADE online tool. The asterisk indicates the conserved amino acids (a.a.) among all proteins.

Supplementary Figure 6

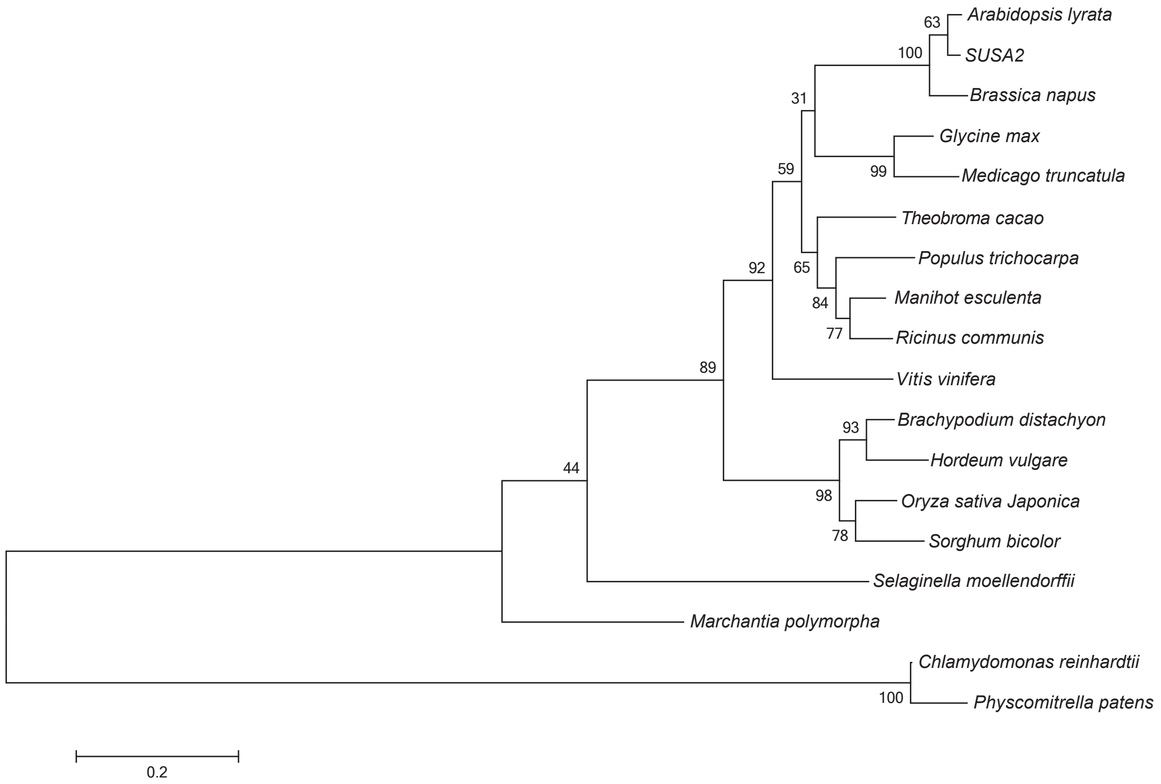

Supplementary Figure 6. Phylogenetic relationship between SUSA2 and its orthologs from multiple plant species.

The protein sequences of SUSA2 and its orthologs in higher plant species were obtained from NCBI protein database using SUSA2 protein sequence as input. Sequences were aligned using Clustal1.83 and a maximum likelihood tree was constructed using Mega6.0 with bootstrap value=1000. Input sequences are: *Brachypodium distachyon* XP_010240578.1, *Hordeum vulgare* BAJ88509.1, *Oryza sativa* XP_015635591.1, *Sorghum bicolor* XP_002447287.1, *Arabidopsis lyrata* XP_020885706.1, *Manihot esculenta* XP_021613734.1, *Ricinus communis* XP_015582439.1, *Populus trichocarpa* XP_002300588.2, *Theobroma cacao* XP_007020318.1, *Glycine max* XP_003522829.1, *Medicago truncatula* XP_003598309.1, *Vitis vinifera* XP_002264128.1, *Selaginella moellendorffii* XP_002968105.1, *Chlamydomonas reinhardtii* XP_001699068.1, *Physcomitrella patens* XP_024390544.1, *Marchantia polymorpha* PTQ30759.1.

Supplementary Figure 7

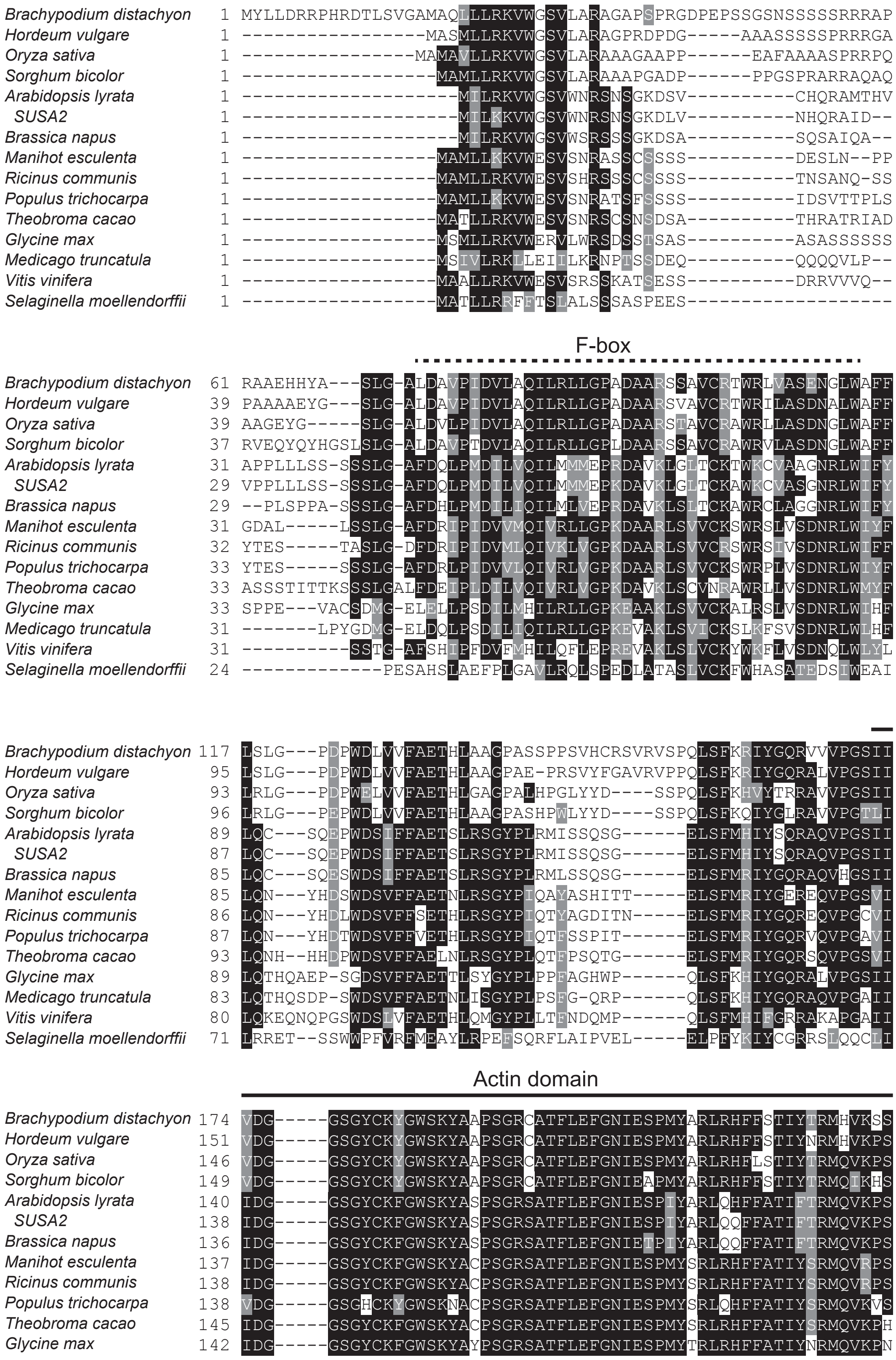

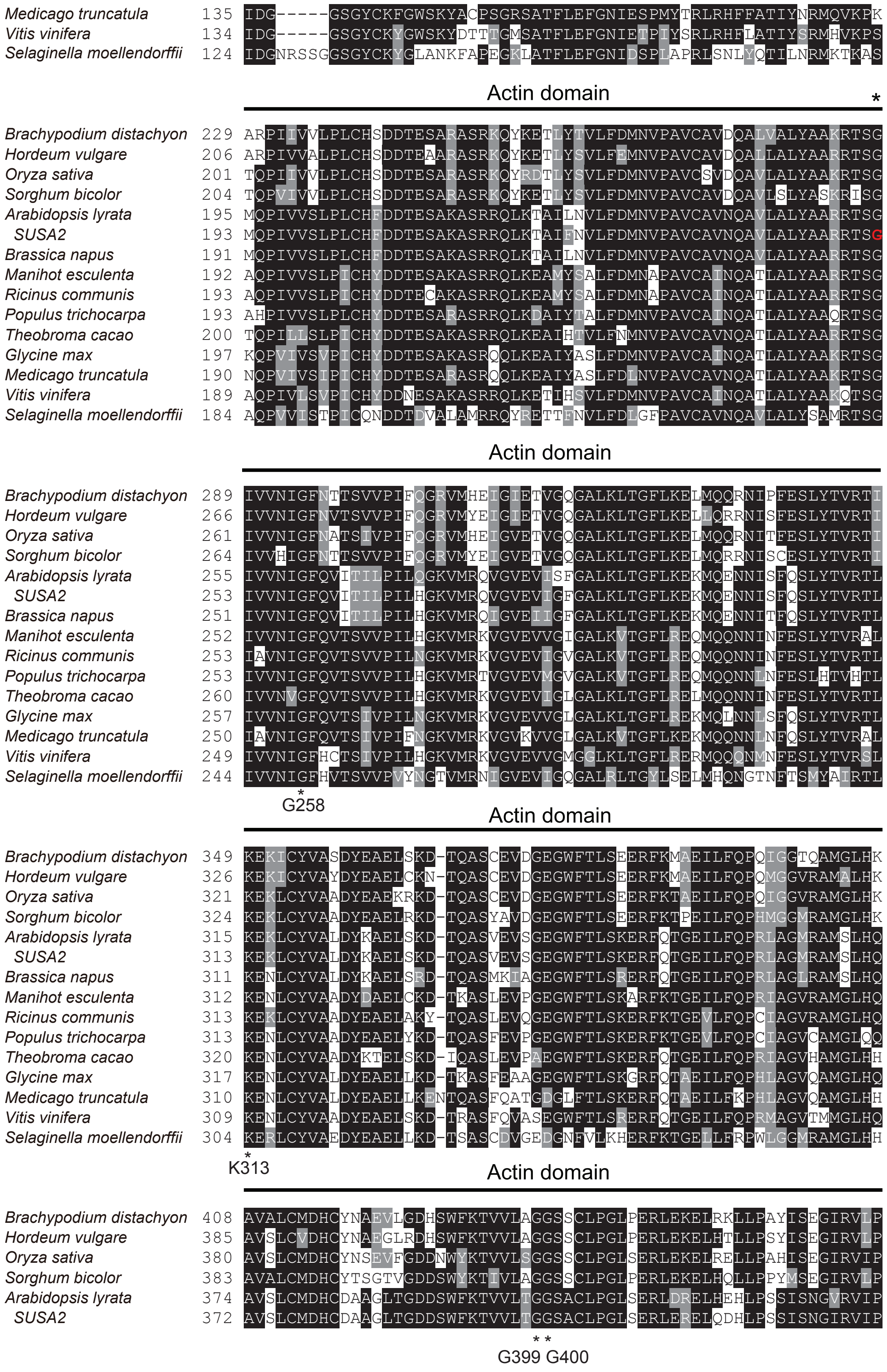

Medicago truncatula 135 IDG-----GSGYCKFGWSKYACPSGRSATFLEFGNIESPMYTRLRHFFATIYNRMQVKPK
Vitis vinifera 134 IDG-----GSGYCKYGWSKYDTTTGMSATFLEFGNIETPIYSRLRHFLATIYSRMHVKPS
Selaginella moellendorffii 124 IDGNRSSGGSGYCKYGLANKFAPEGKLATFLEFGNIDSPLAPRLSNLYQTILNRMKTKAS
Actin domain
*
Brachypodium distachyon 229 ARPIIVVLPLCHSDDTESARASRKQYKETLYTVLFDMNVPAVCAVDQALVALYAAKRTSG
Hordeum vulgare 206 ARPIVVALPLCHSDDTEAARASRKQYKETLYSVLFEMNVPAVCAVDQALLALYAARRTSG
Oryza sativa 201 TQPIIVVLPLCHSDDTESARASRKQYRDTLYSVLFDMNVPAVCSVDQAVLALYAAKRTSG
Sorghum bicolor 204 TQPVIVVLPLCHSDDTESARASRKQYKETLYSVLFDMNVPAVCAVDQAVLSLYASKRISG
Arabidopsis lyrata 195 MQPIVVSLPLCHFDDTESAKASRRQLKTAILNVLFDMNVPAVCAVNQAVLALYAARRTSG
SUSA2 193 MQPIVVSLPLCHFDDTESAKASRRQLKTAIFNVLFDMNVPAVCAVNQAVLALYAARRTSG
Brassica napus 191 MQPIVVSLPLCHFDDTESAKASRRQLKTAILNVLFDMNVPAVCAVNQAVLALYAARRTSG
Manihot esculenta 192 AQPIVVSLPICHYDDTESAKASRRQLKEAMYSALFDMNAPAVCAINQATLALYAARRTSG
Ricinus communis 193 AQPIVVSLPICHYDDTECAKASRRQLKEAMYSALFDMNAPAVCAINQATLALYAARRTSG
Populus trichocarpa 193 AHPIVVSLPLCHYDDTESARASRRQLKDAIYTALFDMNVPAVCAINQATLALYAAQRTSG
Theobroma cacao 200 TQPILLSLPICHYDDTESAKASRRQLKEAIHTVLFNMNVPAVCAVNQATLALYAARRTSG
Glycine max 197 KQPVIVSVPICHYDDTESAKASRQQLKEAIYASLFDMNVPAVCAINQATLALYAAKRTSG
Medicago truncatula 190 NQPVIVSIPICHYDDTESARASRQQLKEAIYASLFDLNVPAVCAVNQATLALYAAKRTSG
Vitis vinifera 189 AQPIVLSVPICHYDDNESAKASRRQLKETIHSVLFDMNVPAVCAINQATLALYAAKQTSG
Selaginella moellendorffii 184 AQPVVISTPICQNDDTDVALAMRRQYRETTFNVLFDLGFPAVCAVNQAVLALYSAMRTSG
Actin domain
Brachypodium distachyon 289 IVVNIGFNTTSVVPIFQGRVMHEIGIETVGQGALKLTGFLKELMQQRNIPFESLYTVRTI
Hordeum vulgare 266 IVVNIGFNVTSVVPIFQGRVMYEIGIETVGQGALKLTGFLKELLQRRNISFESLYTVRTI
Oryza sativa 261 IVVNIGFNATSIVPIFQGRVMHEIGVETVGQGALKLTGFLKELMQQRNITFESLYTVRTI
Sorghum bicolor 264 IVVHIGFNTTSVVPIFQGRVMYEIGVETVGQGALKLTGFLKELMQRRNISCESLYTVRTI
Arabidopsis lyrata 255 IVVNIGFQVITILPILQGKVMRQVGVEVISFGALKLTGFLKEKMQENNISFQSLYTVRTL
SUSA2 253 IVVNIGFQVITILPILHGKVMRQVGVEVIGFGALKLTGFLKEKMQENNISFQSLYTVRTL
Brassica napus 251 IVVNIGFQVITILPILHGKVMRQIGVEIIGFGALKLTGFLKEKMQENNITFQSLYTVRTL
Manihot esculenta 252 IVVNIGFQVTSVVPILHGKVMRKVGVEVVGIGALKVTGFLREQMQQNNINFESLYTVRTL
Ricinus communis 253 IAVNIGFQVTSVVPILNGKVMRKVGVEVIGVGALKVTGFLREQMQQNNINFESLYTVRAL
Populus trichocarpa 253 IVVNIGFQVTSVVPILHGKVMRTVGVEVMGVGALKVTGFLREQMQQNNLNFESLHTVHTL
Theobroma cacao 260 IVVNVGFQVTSVVPILHGKVMRKVGVEVIGLGALKLTGFLRELMQQNNINFESLYTVRTL
Glycine max 257 IVVNIGFQVTSIVPILNGKVMRKVGVEVVGLGALKLTGFLREKMQLNNISFQSLYTVRTL
Medicago truncatula 250 IAVNIGFQVTSIVPIFNGKVMRKVGVKVVGLGALKVTGFLKEKMQQNNLNFQSLYTVRAL
Vitis vinifera 249 IVVNIGFHCTSIVPILHGKVMRKVGVEVVGMGGLKLTGFLRERMQQQNMNFESLYTVRSL
Selaginella moellendorffii 244 IVVNIGFHVTSVVPVYNGTVMRNIGVEVIGQGALRLTGYLSELMHQNGTNFTSMYAIRTL
*
G258
Actin domain
Brachypodium distachyon 349 KEKICYVASDYEAELSKD-TQASCEVDGEGWFTLSEERFKMAEILFQPQIGGTQAMGLHK
Hordeum vulgare 326 KEKICYVAYDYEAELCKN-TQASCEVDGEGWFTLSEERFKMAEILFQPQMGGVRAMALHK
Oryza sativa 321 KEKLCYVAADYEAEKRKD-TQASCEVDGEGWFTLSEERFKTAEILFQPQIGGVRAMGLHK
Sorghum bicolor 324 KEKLCYVAADYEAELRKD-TQASYAVDGEGWFTLSEERFKTPEILFQPHMGGMRAMGLHK
Arabidopsis lyrata 315 KEKLCYVALDYKAELSKD-TQASVEVSGEGWFTLSKERFQTGEILFQPRLAGMRAMSLHQ
SUSA2 313 KEKLCYVALDYKAELSKD-TQASVEVSGEGWFTLSKERFQTGEILFQPRLAGMRAMSLHQ
Brassica napus 311 KENLCYVALDYKAELSRD-TQASMKIAGEGWFTLSRERFQTGEILFQPRLAGLRAMSLHQ
Manihot esculenta 312 KENLCYVAADYDAELCKD-TKASLEVPGEGWFTLSKARFKTGEILFQPRIAGVRAMGLHQ
Ricinus communis 313 KEKLCYVAADYEAELAKY-TQASLEVQGEGWFTLSKERFKTGEVLFQPCIAGVRAMGLHQ
Populus trichocarpa 313 KENLCYVAADYEAELYKD-TQASFEVPGEGWFTLSKERFKTGEVLFQPCIAGVCAMGLQQ
Theobroma cacao 320 KENLCYVAADYKTELSKD-IQASLEVPAEGWFTLSKERFQTGEILFQPRIAGVHAMGLHH
Glycine max 317 KENLCYVALDYEAELLKD-TKASFEAAGEGWFTLSKGRFQTAEILFQPHLAGVQAMGLHQ
Medicago truncatula 310 KENLCYVALDYEAELLKENTQASFQATGDGLFTLSKERFQTAEILFKPHLAGVQAMGLHQ
Vitis vinifera 309 KENLCYVAADYEAELSKD-TRASFQVASEGWFTLSRERFQTGEILFQPRMAGVTMMGLHQ
Selaginella moellendorffii 304 KERLCYVAEDYEAELLKD-TSASCDVGEDGNFVLKHERFKTGEILFRPWLGGMRAMGLHH
*
K313
Actin domain
Brachypodium distachyon 408 AVALCMDHCYNAEVLGDHSWFKTVVLAGGSSCLPGLPERLEKELRKLLPAYISEGIRVLP
Hordeum vulgare 385 AVSLCVDHCYNAEGLRDHSWFKTVVLAGGSSCLPGLPERLEKELHTLLPSYISEGIRVLP
Oryza sativa 380 AVSLCMDHCYNSEVFGDDNWYKTVVLSGGSSCLPGLSERLEKELRELLPAHISEGIRVIP
Sorghum bicolor 383 AVALCMDHCYTSGTVGDDSWYKTIVLAGGSSCLPGLPERLEKELHQLLPPYMSEGIRVLP
Arabidopsis lyrata 374 AVSLCMDHCDAAGLTGDDSWFKTVVLTGGSACLPGLSERLDRELHEHLPSSISNGVRVIP
SUSA2 372 AVSLCMDHCDAAGLTGDDSWFKTVVLTGGSACLPGLSERLERELQDHLPSSISNGIRVIP
* *
G399 G400

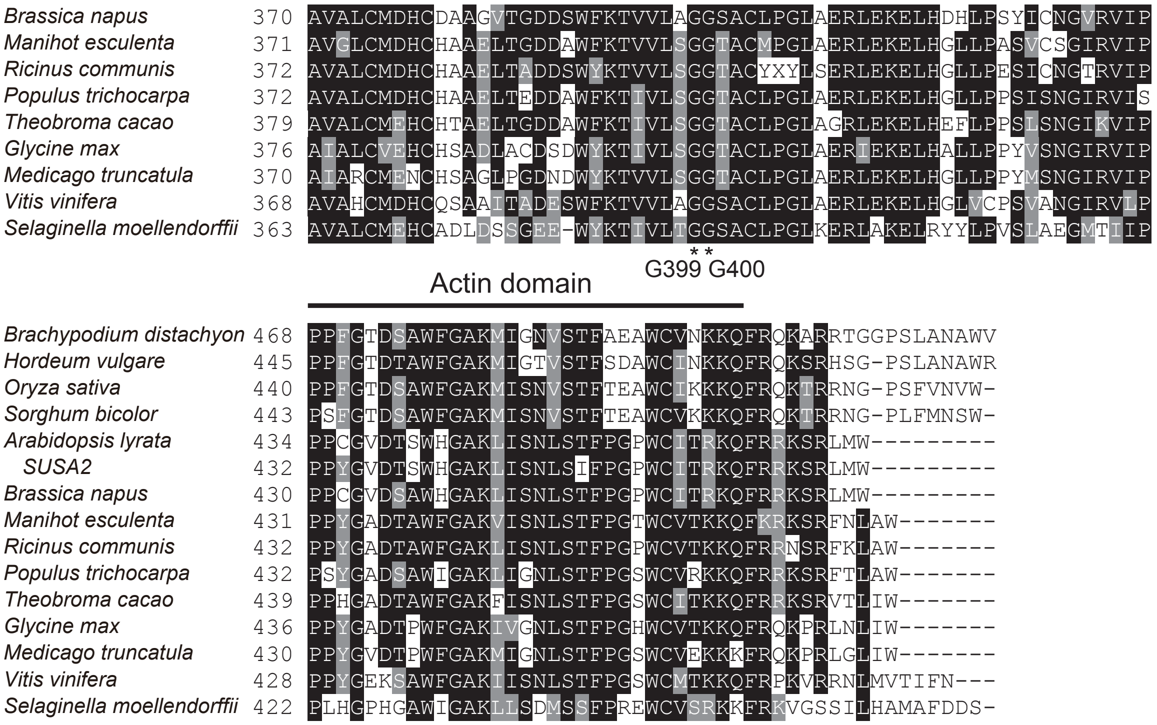

Supplementary Figure 7. Amino acid alignment of SUSA2 and its orthologs from multiple plant species.

The protein sequences of SUSA2 and its orthologs in higher plant species were obtained from NCBI protein database using SUSA2 protein sequence as input. Sequences were aligned using Clustal1.83 and shaded with BOXSHADE online tool. The F-box and ACTIN domains are indicated with dotted and solid black lines, respectively. The asterisk indicates the amino acid (a.a.) change in *susa2-1* mutant.

Supplementary Figure 8

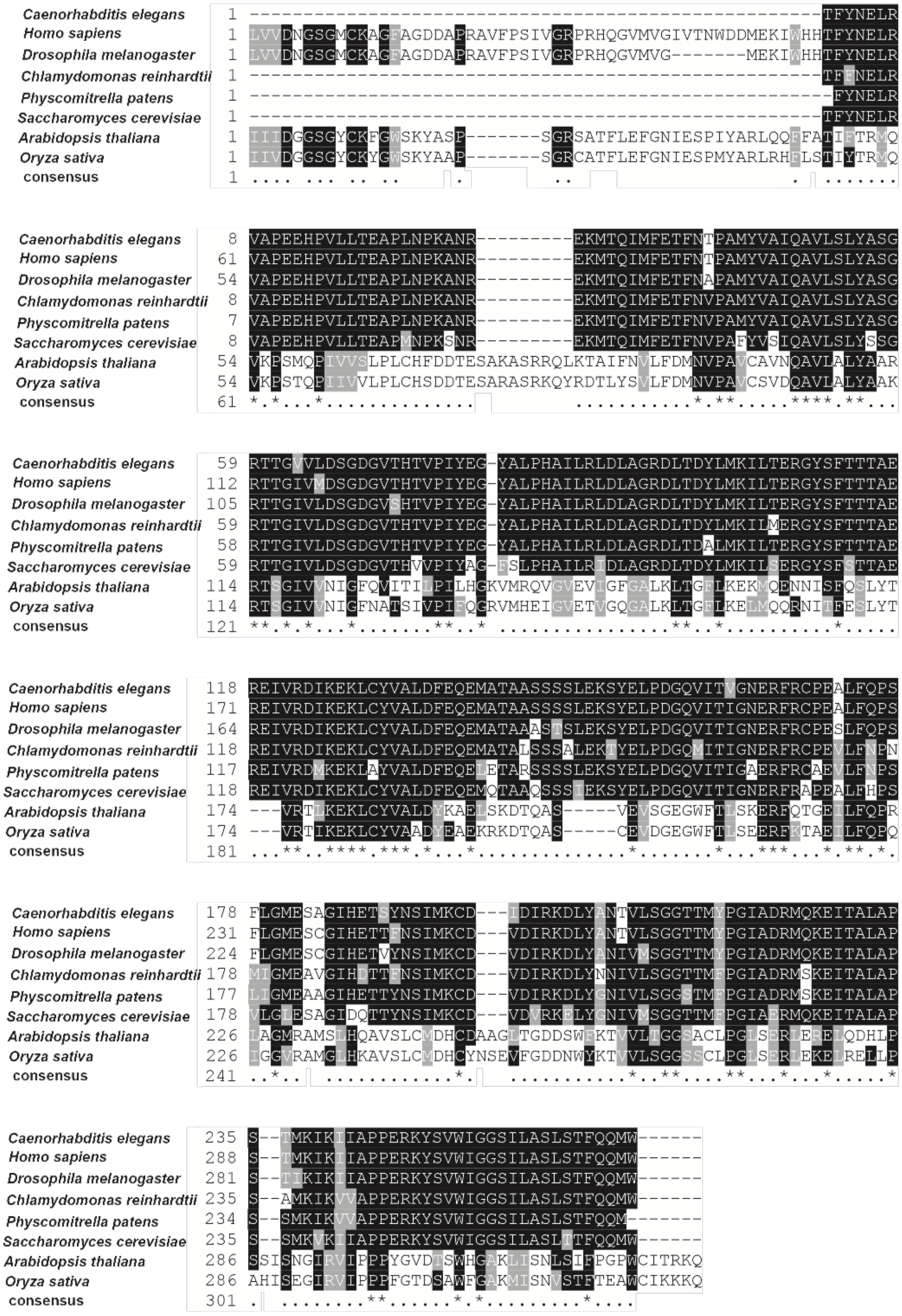

Supplementary Figure 8. Amino acid alignment of ACTIN domain from SUSA2 and its orthologs.

The protein sequences of ACTIN domains from SUSA2 and its orthologs were obtained from NCBI protein database using SUSA2 ACTIN domain protein sequence as input. Sequences were aligned using Clustal1.83 and shaded with BOXSHADE online tool. The asterisk indicates the conserved amino acids (a.a.) among all proteins.

Supplementary Figure 9

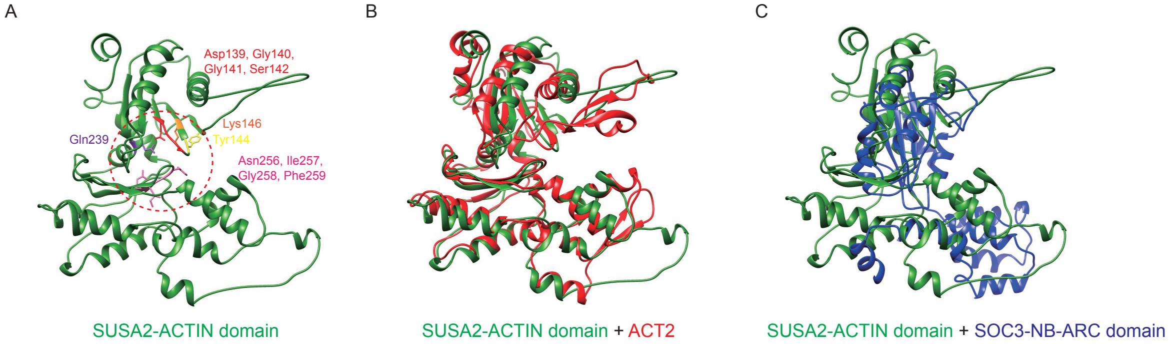

Supplementary Figure 9. Superimposition of the SUSA2 predicted structural model on that of ACT2 and SOC3 NB-ARC domains.

Predicated structural models of SUSA2 ACTIN domain, ACT2 and SOC3 NB-ARC domain were generated using Phyre2 web portal (S10A, S10B and S10C) [74]. Structural model comparisons were performed using software UCSF Chimera (S10B and S10C). The structural similarity between models is indicated by the TM score 0.81, indicating a very high structural similarity between SUSA2 ACTIN domain and ACT2. The red dotted circle in (A) shows the nucleotide binding area of SUSA2 protein with predicated residues indicated (Asp139, Gly140, Gly141, Ser142, Tyr144, Lys146, Gln239, Asn256, Ile257, Gly258 and Phe259).

Supplementary Figure 10

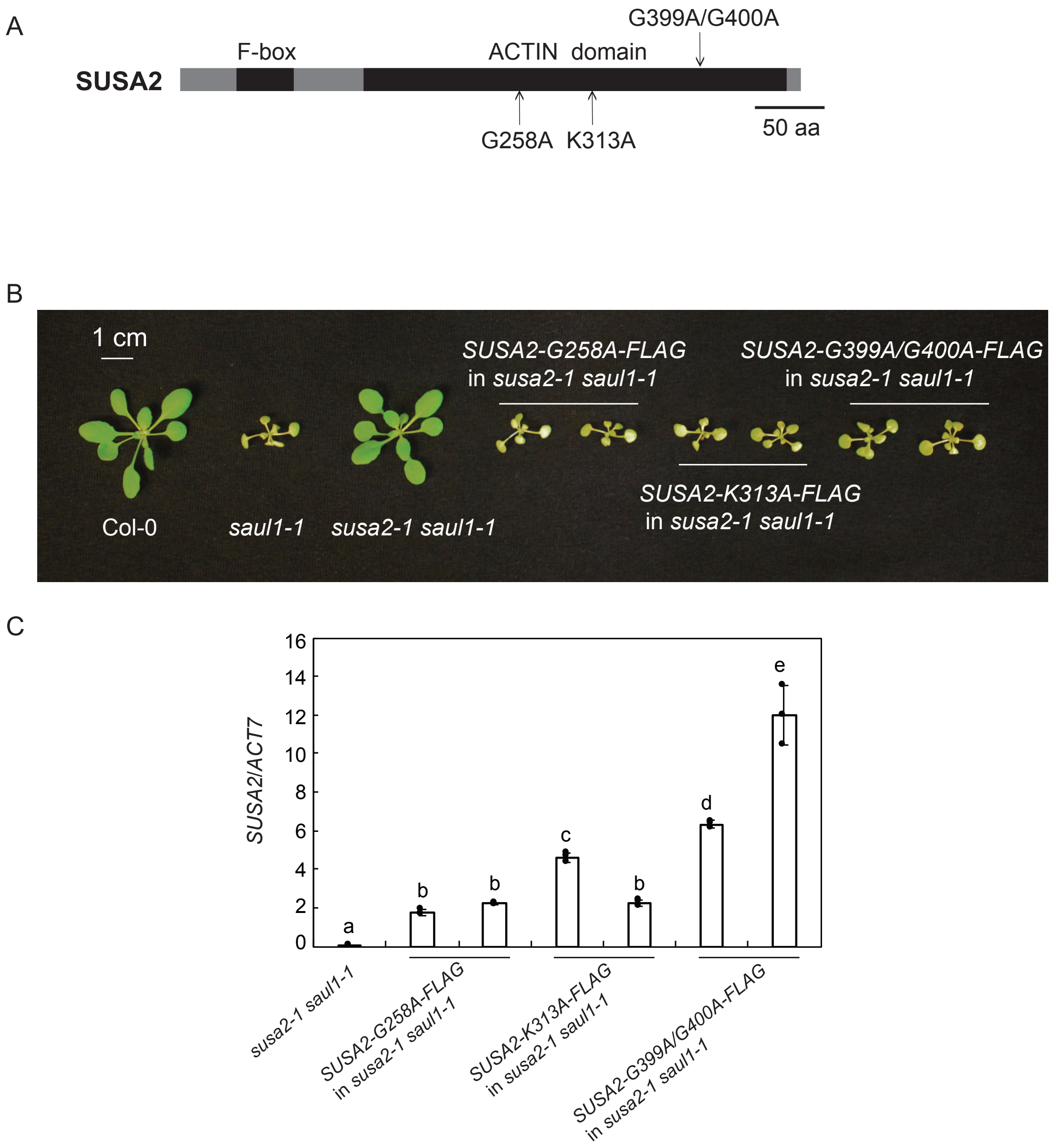

Supplementary Figure 10. Mutant *SUSA2* genes with amino acid substitutions in SUSA2 ACTIN domain still complement *susa2-1 saul1-1* mutant phenotype.

(A) Position of substituted residues in SUSA2 ACTIN domain. The N-terminal F-box domain and C-terminal ACTIN domain are indicated with black boxes. The asterisks indicate the position of substituted residues in SUSA2 ACTIN domain.

(B) Morphology of 3.5-week-old Col-0, *saul1-1*, *susa2-1 saul1-1*, and two independent transgenic plants with SUSA2-FLAG and SUSA2-G258A-FLAG, SUSA2-K313A-FLAG, SUSA2-

G399A/G400A-FLAG transformed into *susa2-1 saul1-1*. Plants were grown on 1/2 MS medium for 10 days and then transplanted to soil for 2 weeks before the picture was taken.

(C) Expression level of *SUSA2* in the indicated plants as determined by RT-PCR and normalized to *ACT7* gene. Error bars represent means ±SD (One-way ANOVA, SPSS Statistics, n=3, $p<0.01$). Experiments were repeated three times with similar results.

Supplementary Figure 11

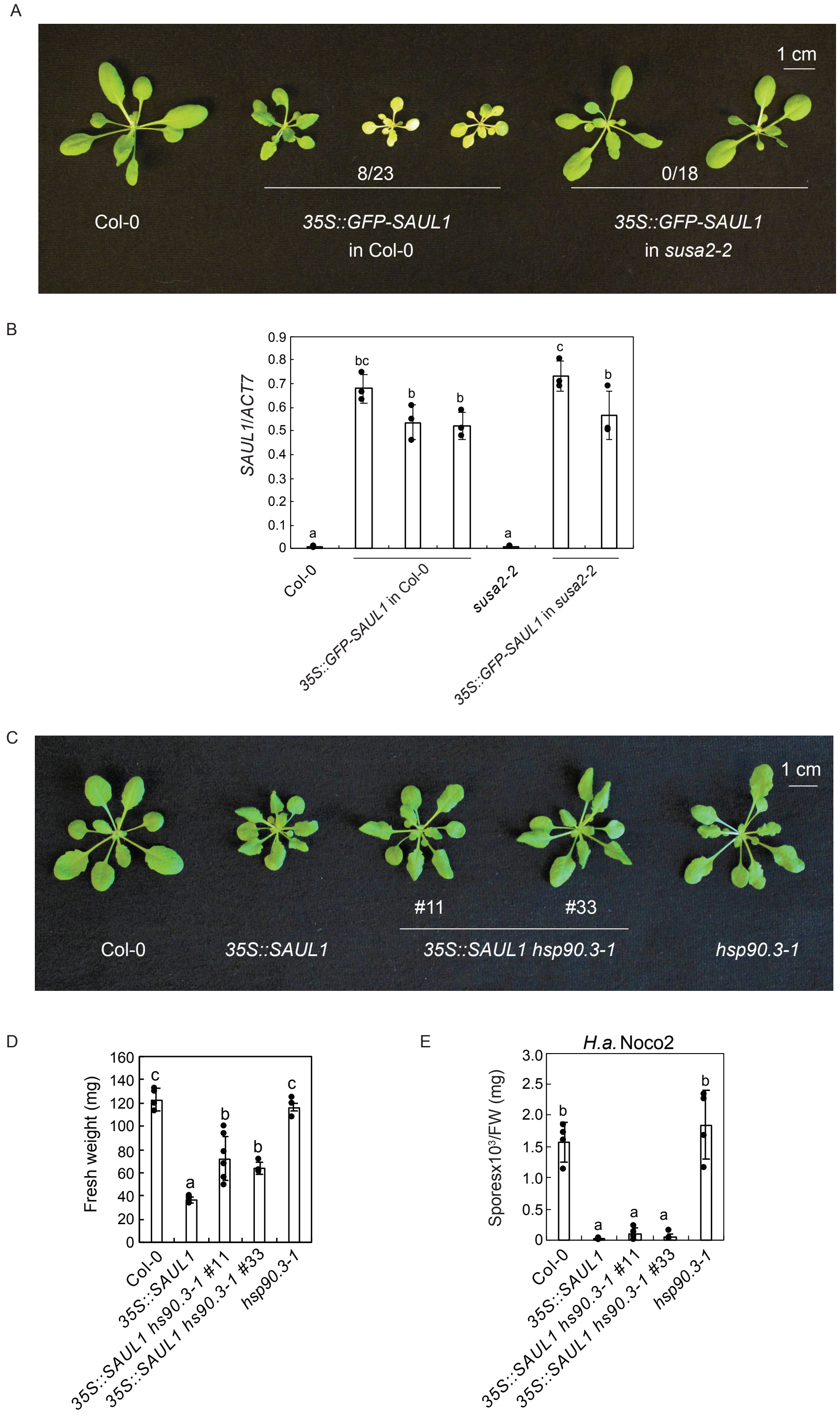

Supplementary Figure 11. SUSA2 and HSP90.3 are required for the *SAUL1* overexpression phenotypes.

(A) Morphology of 4-week-old Col-0, three representative *35S::GFP-SAUL1* in Col-0 T1 plants with curly leaves and cell death phenotype, two representative *35S::GFP-SAUL1* in *susa2-2* T1 plants. 8/23 means 8 out of 23 T1 transformants of *SAUL1* overexpression in Col-0 exhibited autoimmunity, including curly leaves and necrosis. 0/18 means none of 18 T1 transformants of *SAUL1* overexpression displayed autoimmunity.

(B) Expression level of *SAUL1* in the indicated plants as determined by RT-PCR and normalized to *ACT7* gene. Error bars represent means ±SD (One-way ANOVA, SPSS Statistics, n=3, $p<0.01$). Experiments were repeated three times with similar results.

(C) Morphology of 4-week-old Col-0, *35S::SAUL1*, *35S::SAUL1 hsp90.3-1* double mutant #11, #33 and *hsp90.3-1*.

(D) Quantification of fresh weight (mg) per plant of the indicated genotypes. Error bars represent means ±SD (One-way ANOVA, SPSS Statistics, n=4, 5, 7, 3, 3 for Col-0, *35S::SAUL1*, *35S::SAUL1 hsp90.3-1* double mutant #11, #33 and *hsp90.3-1*, respectively, $p<0.01$). Experiments were repeated three times with similar results.

(E) Quantification of oomycete pathogen *H.a.* Noco2 growth on the indicated genotypes. Three-week-old plants were evenly sprayed with *H.a.* Noco2 conidiospores at a concentration of 100,000 spores/ml water. Quantification of conidia growth on leaf surface per mg FW was determined 7 days post inoculation (dpi). Error bars represent means ±SD (One-way ANOVA, SPSS Statistics, n=4, $p<0.01$). Experiments were repeated three times with similar results.

Supplementary Figure 12

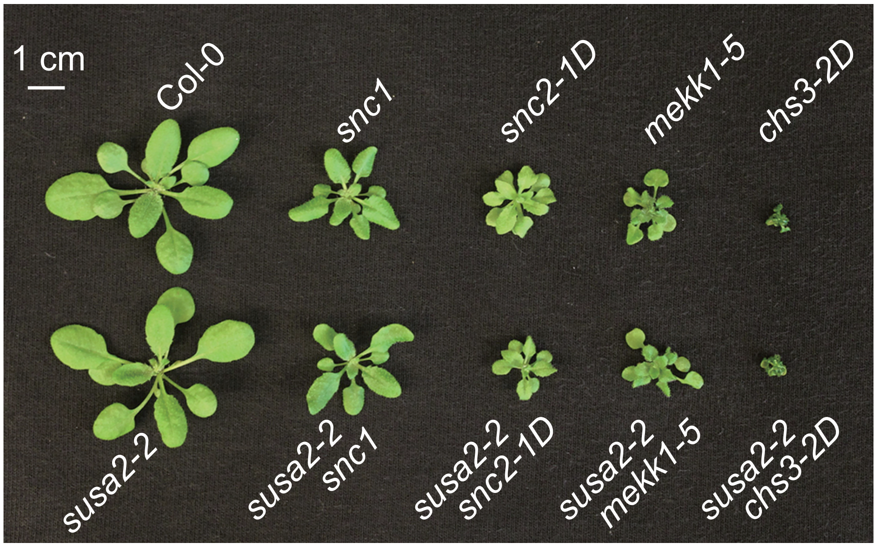

Supplementary Figure 12. *susa2-2* cannot suppress the autoimmunity of *snc1*, *snc2-1D*, *mekk1-5* or *chs3-2D*.

Morphology of four-week-old Col-0, *snc1*, *snc2-1D*, *mekk1-5*, *chs3-2D*, *susa2-2* and double mutants of *susa2-2* with *snc1*, *snc2-1D*, *mekk1-5*, or *chs3-2D*.

Supplementary Figure 13

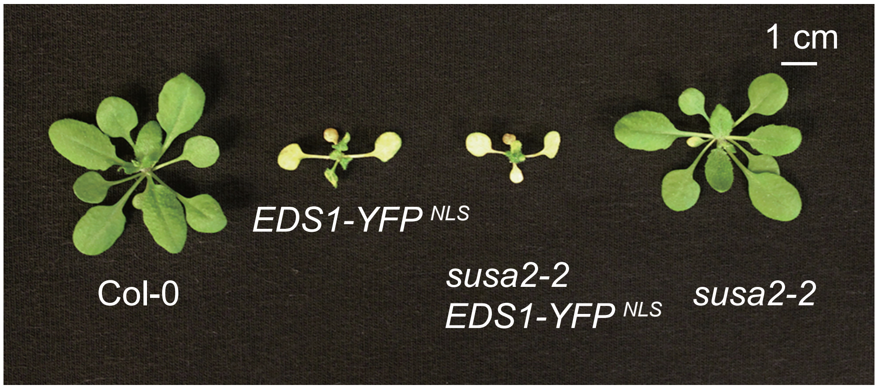

Supplementary Figure 13. Epistasis analysis between *susa2-2* and *EDS1-YFP^{NLS}*. Morphology of four-week-old Col-0, *EDS1-YFP^{NLS}*, *EDS1-YFP^{NLS} susa2-2* double mutant and *susa2-2* plants.

Supplementary Figure 14

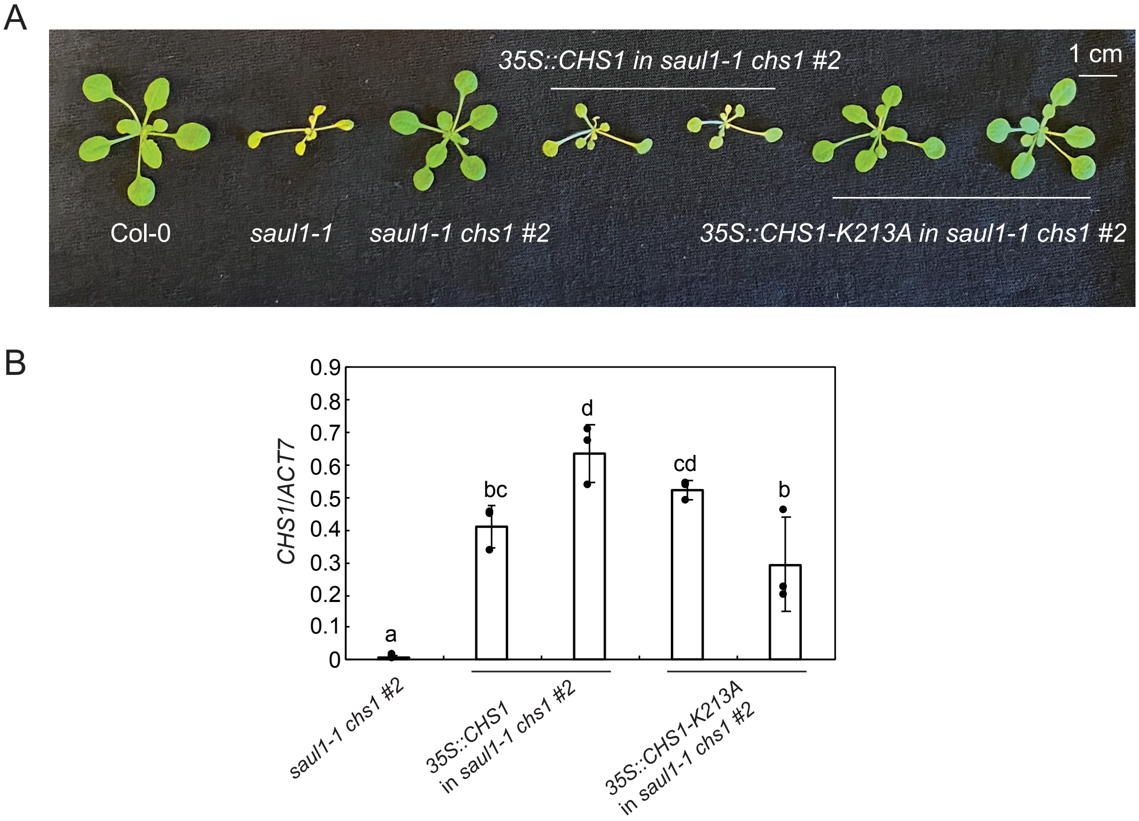

Supplementary Figure 14. CHS1 P-loop is required for *saul1-1* autoimmunity.

(A) Morphology of four-week-old Col-0, *saul1-1*, *saul1-1 chs1* #2, two independent transgenic plants with *35S::CHS1* transformed into *saul1-1 chs1* #2, and two independent transgenic plants with *35S::CHS1-K213A* (P-loop mutation) transformed into *der1-1*.

(B) Expression level of *CHS1* in the indicated plants as determined by RT-PCR and normalized to *ACT7* gene. Error bars represent means ±SD (One-way ANOVA, SPSS Statistics, n=3, $p<0.01$). Experiments were repeated three times with similar results.

Supplementary Figure 15

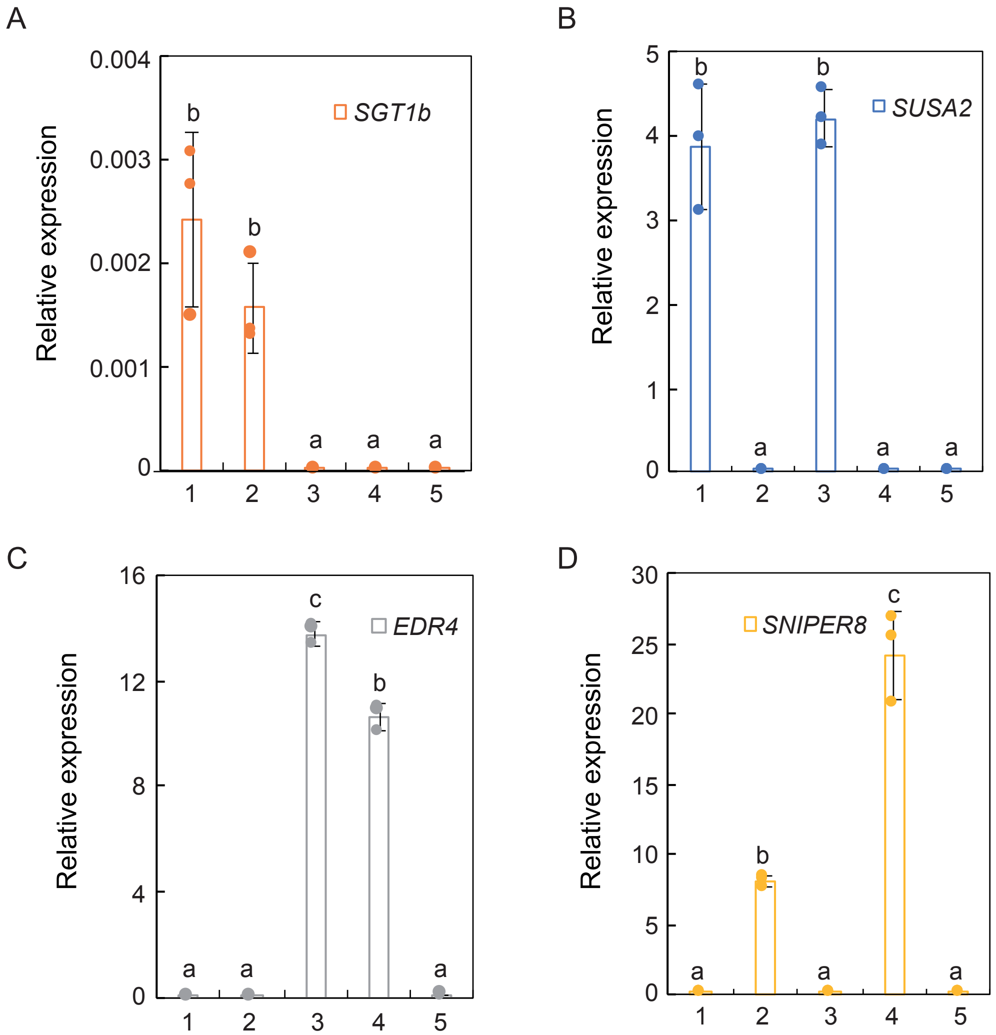

Supplementary Figure 15. Expression level of *SGT1b*, *SUSA2*, *EDR4* and *SNIPER8* in SUSA2-SGT1b split-luciferase assay (related to Figure 6B).

(A)-(D) Expression level of *SGT1b*, *SUSA2*, *EDR4* and *SNIPER8* in the indicated infiltrated area in (Figure 6B) as determined by RT-PCR and normalized to *NbACTIN* gene. Error bars represent means ±SD (One-way ANOVA, SPSS Statistics, n=3, p<0.01). Experiments were repeated three times with similar results.

Supplementary Tables

Supplementary Table 1

Plasmid/purpose	Primer name	sequence
SUSA2::SUSA2	SUSA2-EcoRI_F	CCGGAATTCTTACGCTTACCGCAAAATTCACTTC
	SUSA2-KpnI_R	CGGGGTACCTCACCACATGAGTCTTGACTTG
SUSA2-FLAG, SUSA2-HA	SUSA2-KpnI_F	CGGGGTACCATGATCCTGAAGAAAGTATGGGGA
	SUSA2-BamHI _R	CGCGGATCCCCACATGAGTCTTGACTTGCG
DN-SUSA2-NLuc, SUSA2-NLuc	SUSA2-KpnI_F	CGGGGTACCATGATCCTGAAGAAAGTATGGGGA
	SUSA2-BamHI _R	CGCGGATCCCCACATGAGTCTTGACTTGCG
DN-SUSA2-Turbo-HA	SUSA2-KpnI_F	CGGGGTACCATGATCCTGAAGAAAGTATGGGGA
	SUSA2-BamHI _R	CGCGGATCCCCACATGAGTCTTGACTTGCG
DN-SUSA2-FLAG	SUSA2-F-box-dele_F	CTCTTGGTGCGTTTGATCAGCTCCAGTGTTCTCAAGAGCC
	SUSA2-F-box-dele_R	GGCTCTTGAGAACACTGGAGCTGATCAAACGCACCAAGAG
SUSA2-G258A-FLAG	SUSA2_G258A_F	TTGTTGTCAACATTGCTTTCCAAGTCATCA
	SUSA2_G258A_R	TGATGACTTGGAAAGCAATGTTGACAACAA
SUSA2-K313A-FLAG	SUSA2_K313A_F	CTGTTCGTACTCTTGCAGAGGTATGACCAT
	SUSA2_K313A_R	ATGGTCATACCTCTGCAAGAGTACGAACAG
SUSA2-G399A/G400A-FLAG	SUSA2_G399A/G400A_F	GTAGTACTAACTGCGGCAAGCGCGTGTTTG
	SUSA2_G399A/G400A_R	CAAACACGCGCTTGCCGCAGTTAGTACTAC
SUSA2 RT-PCR	SUSA2-RT-F	GGTGATGCGCCAGGTAGGTG
	SUSA2-RT-R	ACGCGCTTCCCCCAGTTAG
susa2-1 genotyping	SUSA2-seq_F	TTCCACGGTTTAAGCAAGTAG
	*susa2-1*_EcoRI_R	ACTTGGAAACCAATGTTGACAAGAATT
susa2-2/SALK_093650 genotyping	*susa2-2*_LP	TTTCACCAGGTTTGTTTTTCG
	*susa2-2*_RP	CGGAAGAGGAATTTAGGATGG
susa2-3/SALK_020877 genotyping	SUSA2-seq_F	TTCCACGGTTTAAGCAAGTAG
	*susa2-2*_RP	CGGAAGAGGAATTTAGGATGG
saul1-1 genotyping	*saul1-1*_LP	ACTCGGAAGCCTTGTTGCTA
	*saul1-1*_RP	TGAGGCCAATCAAATGATTTC
CS878039 genotyping	CS878039_LP	CGAGAAGCACAACTCCGTAAC
	CS878039_RP	TCGTTATCGTTTGGTTCTTGG

SALK_020826 genotyping	SALK_020826_LP	GGCCACTCATACTAGTCCTGC
	SALK_020826_RP	CTTAACATTCTTCTTCCCGGC
CHS1-Turbo-HA	CHS1-KpnI_F	CGGGGTACCATGTCTACTTCTTATTCTTTTTTGTTGGCT
	CHS1-SpeI _R	CGGACTAGTTCTTTGGGATGCTTCCACG
CHS1-HA	CHS1-BamHI_F	CGCGGATCCATGTCTACTTCTTATTCTTTTTTGTTGGCT
	CHS1-SalI _R	ACGCACGCGTCGACTCTTTGGGATGCTTCCACG
CHS1 RT-PCR	Ref [24]	
35S::CHS1	CHS1-KpnI_F	CGGGGTACCATGTCTACTTCTTATTCTTTTTTGTTGGCT
	CHS1-SpeI _R	CGGACTAGT TCTTTGGGATGCTTCCACG
35S::CHS1-K213A	CHS1-KpnI_F	CGGGGTACCATGTCTACTTCTTATTCTTTTTTGTTGGCT
	CHS1-SpeI _R	CGGACTAGT TCTTTGGGATGCTTCCACG
	CHS1- P-loop-K213A_F	GGGTAGTTCAGGTGTGGGAGCGACAACACTTGCAAGGTAC
	CHS1- P-loop-K213A_R	GTACCTTGCAAGTGTTGTCGCTCCCACACCTGAACTACCC
TN2-FLAGTEVZZ	TN2-KpnI _F	GGGGTACCATGTATTCATCATCGTCTTCTTCTTCAG
	TN2-PstI_R	AACTGCAGAGAAGATTCAGTCCCGGATATAGG
TN2-HA	TN2-XbaI _F	GCTCTAGA ATGTATTCATCATCGTCTTCTTCTTCAG
	TN2-PstI_R	AACTGCAGAGAAGATTCAGTCCCGGATATAGG
HA-FLAG-SOC3	Ref [25]	
SGT1b-CLuc	SGT1B-KpnI _F	CGGGGTACCATGGCCAAGGAATTAGCAGAG
	SGT1B-SpeI_R	CGGACTAGTATACTCCCACTTCTTGAGCT
SGT1b RT-PCR	SGT1b-RT_F	ATGGCCAAGGAATTAGCAGAG
	SGT1b-RT_R	TCAATACTCCCACTTCTTGAGCT
HSP90.3-HA	HSP90.3-SpeI_F	CGGACTAGTATGGCGGACGCAGAAACCTTTG
	HSP90.3-PstI_R	AACTGCAGGTCAACTTCCTCCATCTTGC
HSP90.3::HSP90.3	Ref [37]	
HSP90.3 RT-PCR	Ref [37]	
hsp90.3-1 genotyping	Ref [37]	
ASK1-CLuc	ASK1-BamHI_F	CGCGGATCCATGTCTGCGAAGAAGATTGTGTTG
ASK1-CLuc	ASK1-SpeI_R	CGGACTAGTTTCAAAAGCCCATTGGTTCTCT
ASK1-FLAGTEVZZ, ASK1-HA	ASK1-KpnI_F	CGGGGTACCATGTCTGCGAAGAAGATTGTGTTG
	ASK1-PstI_R	AACTGCAGTTCAAAAGCCCATTGGTTCTCT
ASK1 RT-PCR	ASK1-RT_F	ATGTCTGCGAAGAAGATTGTGTTG
	ASK1-RT_R	TTCAAAAGCCCATTGGTTCTCT

35S::GFP-SAUL1	Ref [25]	
HA-SAUL1	Ref [25]	
SAUL1-C29A-FLAG	Ref [25]	
SAUL1 RT-PCR	SAUL1-RT_F	GAGAGCAAGGCGATAGTAGC
	SAUL1-RT_R	TCAGGTGAACCTTCAAGGAG
EDR4-Cluc	EDR4_KpnI_F	CGGGGTACCATGGCGAGCCAGACGGGTCA
	EDR4_SalI_R	ACGCACGCGTCGACTATAGAAGGAGGACGCTGTGAAA
EDR4 RT-PCR	EDR4-RT_F	ATGGCGGTTGGTATTCAGGTCA
	EDR4-RT_R	GGTGGCCAGCATGTCTATTACG
