# Supplementary material for: SUSA2 is an F-box protein required for autoimmunity mediated by paired NLRs SOC3-CHS1 and SOC3-TN2
Source: Nat Commun. 2020 Oct 15;11:5190. doi: 10.1038/s41467-020-19033-z (PMC7562919; doi:10.1038/s41467-020-19033-z)
Supplement: Supplementary file 3 — Reporting Summary [file 41467_2020_19033_MOESM3_ESM.pdf]

nature research

Corresponding author(s): Dr. Xin Li

Last updated by author(s): Sep 17, 2020

Reporting Summary

Nature Research wishes to improve the reproducibility of the work that we publish. This form provides structure for consistency and transparency in reporting. For further information on Nature Research policies, see our Editorial Policies and the Editorial Policy Checklist.

Statistics

For all statistical analyses, confirm that the following items are present in the figure legend, table legend, main text, or Methods section.

n/a	Confirmed	
☐	☒	The exact sample size (n) for each experimental group/condition, given as a discrete number and unit of measurement
☐	☒	A statement on whether measurements were taken from distinct samples or whether the same sample was measured repeatedly
☐	☒	The statistical test(s) used AND whether they are one- or two-sided<br>*Only common tests should be described solely by name; describe more complex techniques in the Methods section.*
☒	☐	A description of all covariates tested
☐	☒	A description of any assumptions or corrections, such as tests of normality and adjustment for multiple comparisons
☐	☒	A full description of the statistical parameters including central tendency (e.g. means) or other basic estimates (e.g. regression coefficient) AND variation (e.g. standard deviation) or associated estimates of uncertainty (e.g. confidence intervals)
☐	☒	For null hypothesis testing, the test statistic (e.g. F, t, r) with confidence intervals, effect sizes, degrees of freedom and P value noted<br>*Give P values as exact values whenever suitable.*
☒	☐	For Bayesian analysis, information on the choice of priors and Markov chain Monte Carlo settings
☒	☐	For hierarchical and complex designs, identification of the appropriate level for tests and full reporting of outcomes
☒	☐	Estimates of effect sizes (e.g. Cohen's d, Pearson's r), indicating how they were calculated

Our web collection on statistics for biologists contains articles on many of the points above.

Software and code

Policy information about availability of computer code

Data collection	RT-PCR data was collected using Bio-Rad CFX ConnectTM Real-Time software.<br>Trypan blue staining photos were taken by camera using Dinocapture 2.0 software.<br>The blots and split luciferase fluorescence signal were detected and quantified on a Bio-Rad gel documentation system.<br>DNA and protein sequences were aligned using Clustal1.83 and shaded with BOXSHADE online tool.<br>A maximum likelihood tree was constructed using Mega6.0.<br>Predicated structural models of SUSA2 ACTIN domain, ACT2 and SOC3 NB-ARC domain were generated using Phyre2 web portal.<br>Structural model comparisons were performed using software UCSF Chimera.
Data analysis	Graphs were made using Microsoft Excel. Statistical analysis was performed by SPSS Statistics.

For manuscripts utilizing custom algorithms or software that are central to the research but not yet described in published literature, software must be made available to editors and reviewers. We strongly encourage code deposition in a community repository (e.g. GitHub). See the Nature Research guidelines for submitting code & software for further information.

Data

Policy information about availability of data

All manuscripts must include a data availability statement. This statement should provide the following information, where applicable:

- Accession codes, unique identifiers, or web links for publicly available datasets
- A list of figures that have associated raw data
- A description of any restrictions on data availability

Data availability
The authors declare that the data supporting the findings of this study are available within the article and its Supplementary Information files.

Field-specific reporting

Please select the one below that is the best fit for your research. If you are not sure, read the appropriate sections before making your selection.

☒ Life sciences ☐ Behavioural & social sciences ☐ Ecological, evolutionary & environmental sciences

For a reference copy of the document with all sections, see nature.com/documents/nr-reporting-summary-flat.pdf

Life sciences study design

All studies must disclose on these points even when the disclosure is negative.

Sample size	The sample size was not predetermined by statistical methods but based on common practice and previous studies. For example, ion leakage measurement was performed according to Ishitani et al., 1998. Infection assays were based on protocols from Zhang & Li, 2005 and Zhang et al., 2003.
Data exclusions	No data were excluded from the analysis.
Replication	Three to four replicates were performed for related experiments, e.g. RT-PCR, infection assays, Fresh weight et al. and noted in the figure legends and methods section.
Randomization	For each specific experiment, seeds of different genotypes were planted and grown under the similar growth conditions. For each genotype, 6 plants with similar size and morphology were randomly chosen for infection assays. For MPK activation, seedlings were treated for the same length of time period 15min before harvesting for western blot analysis.
Blinding	Blinding was not possible for us. The reason is that different mutants and transgenic lines were involved in the study.

Reporting for specific materials, systems and methods

We require information from authors about some types of materials, experimental systems and methods used in many studies. Here, indicate whether each material, system or method listed is relevant to your study. If you are not sure if a list item applies to your research, read the appropriate section before selecting a response.

Materials & experimental systems

n/a	Involved in the study	
☐	☒	Antibodies
☒	☐	Eukaryotic cell lines
☒	☐	Palaeontology and archaeology
☒	☐	Animals and other organisms
☒	☐	Human research participants
☒	☐	Clinical data
☒	☐	Dual use research of concern

Methods

n/a	Involved in the study	
☒	☐	ChIP-seq
☒	☐	Flow cytometry
☒	☐	MRI-based neuroimaging

Antibodies

Antibodies used	Anti-HA (11867423001, Roche),<br>Anti-FLAG (Cat. #F1804, Sigma),<br>Anti-Erk antibody (Cell signaling; #4370S),<br>Streptavidin-HRP (Abcam Cat. # ab7403),<br>goat anti-mouse (32230, Thermo Fisher),<br>goat anti-rat (2065, Santa Cruz) ,<br>goat anti-rabbit (2030, Santa Cruz)

Validation	The biological source of Anti-HA (Cat. #11867423001, Roche), Anti-FLAG (Cat. #F1804, Sigma) and Anti-Erk antibody (Cell signaling; #4370S) is rat, mouse and rabbit, respectively. The specificity of the primary antibodies was validated by the manufacturer and in publications listed on the manufacturer website.
